# A Digital Twin of the Angiotensin II Receptor Blocker Losartan: Physiologically Based Modeling of Blood Pressure Regulation

**DOI:** 10.3390/pharmaceutics18020262

**Published:** 2026-02-19

**Authors:** Ennie Tensil, Mariia Myshkina, Matthias König

**Affiliations:** 1 Charité—Universitätsmedizin Berlin, corporate member of Freie Universität Berlin and Humboldt-Universität zu Berlin, Charitéplatz 1, 10117 Berlin, Germany; 2 Faculty of Life Sciences, Department of Biology, Institute of Theoretical Biology, Systems Medicine of the Liver, Humboldt-Universität zu Berlin, Unter den Linden 6, 10099 Berlin, Germany; 3 Institute of Structural Mechanics and Dynamics in Aerospace Engineering, University of Stuttgart, Pfaffenwaldring 27, 70569 Stuttgart, Germany

**Keywords:** losartan, angiotensin II receptor blocker, ARB, PBPK/PD modeling, pharmacokinetics, pharmacodynamics, SBML

## Abstract

**Background/Objectives:** Losartan, an angiotensin II receptor blocker (ARB) used to treat hypertension and heart failure, shows significant variability in pharmacokinetics (PK) and pharmacodynamics (PD) among individuals. **Methods:** In this study, we developed a physiologically based pharmacokinetic/pharmacodynamic (PBPK/PD) model of losartan and its active metabolite, E3174, using curated data from 25 clinical trials. The model mechanistically describes the processes of absorption, hepatic metabolism, renal and fecal excretion, and pharmacodynamic blood pressure regulation. Simulation studies examined the effects of dose, hepatic and renal impairment, and genetic polymorphisms in cytochrome p450 2C9 (CYP2C9) and P-glycoprotein 1, also known as multidrug resistance protein 1 (MDR1) or ATP-binding cassette sub-family B member 1 (ABCB1), on the model. **Results:** The model successfully reproduced key PK/PD observations, including dose-dependent receptor blockade, attenuated responses with hepatic impairment, modest enhancement with renal impairment, and substantial variability in E3174 formation dependent on CYP2C9; the effects of ABCB1 were minimal. Specifically, dose dependency simulations confirmed the saturable nature of CYP2C9 metabolism, predicting a decreasing E3174-to-losartan ratio and a stronger, sustained suppression of blood pressure and aldosterone at higher doses. Hepatic impairment was predicted to lead to elevated losartan plasma concentrations (increased AUC) and attenuated metabolite formation, confirming the clinical need for dose reduction. Renal impairment simulations predicted stable losartan AUC but showed an overestimation of E3174 accumulation compared to observed data, where E3174 exposure remained stable. Genetic variability (CYP2C9) was the major determinant of response, with simulations confirming that reduced-function alleles lead to a 1.6- to 3-fold increase in losartan AUC and diminished blood pressure reduction. ABCB1 variability resulted in only minor modulation of systemic exposure and blood pressure effects. **Conclusions:** This mechanistic digital twin framework provides a quantitative basis for understanding variability in losartan therapy and supports its application in individualized dosing strategies.

## 1. Introduction

Hypertension is a major global health challenge and the leading risk factor for cardiovascular disease, stroke, and kidney failure [1,2,3]. It is defined as a systolic blood pressure of ≥140 mmHg or a diastolic blood pressure of ≥90 mmHg according to the World Health Organization [1] and the European Society of Cardiology (ESC) [4]. In 2019, over 1.2 billion people worldwide were affected, with prevalence exceeding 50% in some regions [2]. Despite its silent clinical course, hypertension contributes to over 8 million deaths annually [3]. Risk factors include age, genetics, obesity, inactivity, and an unhealthy diet [1]. While treatment options exist, improved prevention and personalized therapies remain essential.

The renin–angiotensin–aldosterone system (RAAS) regulates blood pressure and fluid balance. Its core effector is angiotensin II, which promotes an increase in blood pressure via vasoconstriction, aldosterone release, and enhanced sodium reabsorption [5]. Dysregulation of the RAAS underlies hypertension, heart failure, and kidney disease. Pharmacological treatments target RAAS at multiple points, for example, beta-blockers lower renin, direct renin inhibitors (e.g., aliskiren) block its activity, ACE inhibitors (e.g., ramipril) prevent angiotensin II formation, while angiotensin II receptor blockers (ARBs) (e.g., losartan) selectively antagonize angiotensin II receptor type 1 (AT1 receptors) [5,6,7,8]. ARBs offer high specificity by blocking receptor-mediated effects of angiotensin II without interfering with its synthesis.

Losartan, the first angiotensin II receptor blocker (ARB) approved in 1995, has long been used to treat hypertension and its associated complications [9,10]. However, telmisartan or candesartan are now more commonly prescribed, as they are generally considered more effective at controlling blood pressure [11,12]. By competitively blocking AT1 receptors, it reduces vasoconstriction, aldosterone secretion, and water retention. Its major active metabolite, E3174, is generated through CYP2C9-mediated conversion and is 10–40 times more potent [13], though losartan itself remains an effective antagonist. Both compounds contribute to clinical efficacy. Losartan is well tolerated, with dizziness and mild respiratory symptoms as the most common side effects [10]. It is available as monotherapy or combination therapy with hydrochlorothiazide or, more commonly, amlodipine for greater blood pressure reduction [14,15]. Standard dosing ranges from 25 to 100 mg/day. Like other ARBs, it is contraindicated in pregnancy [9].

Losartan shows rapid oral absorption, with peak plasma levels after 1–2 h and bioavailability of about 33% due to first-pass metabolism [13,16]. E3174 peaks later (3–4 h) and has 4–8-fold higher systemic exposure [13,17]. Both compounds bind strongly to plasma proteins and are eliminated via hepatic metabolism and renal/fecal excretion, with terminal half-lives of about 2 h (losartan) and 4–6 h (E3174) [18,19]. Only small fractions are excreted unchanged renally [20]. Minor metabolites such as L158 reflect additional metabolic pathways [19,21].

Pharmacodynamically, losartan lowers blood pressure by blocking AT1 receptors, thereby reducing aldosterone secretion [22,23,24]. AT1 blockade also increases renin and angiotensin levels due to loss of feedback control [25,26]. Enhanced stimulation of angiotensin II receptor type 2 (AT2 receptors) may further contribute to vasodilation [27].

Liver cirrhosis reduces losartan clearance by about 50% and doubles bioavailability, leading to higher plasma levels, though E3174 exposure increases only modestly [9,28]. Renal impairment decreases clearance of losartan and E3174 but generally does not elevate E3174 levels due to compensatory routes or mechanisms of elimination [20,29,30]. The reduced E3174 renal elimination could be compensated by an increase in biliary (fecal) elimination, since this route contributes to the total elimination of losartan and E3174 [12]. Neither compound is dialyzable, and dose adjustments are generally required in hepatic, but not renal impairment [31].

Drug response varies due to genetic polymorphisms in the ABCB1 and CYP2C9 genes. Variants of ABCB1, a gene encoding for a drug efflux transporter also known as P-glycoprotein or MDR1, influence losartan absorption and blood pressure response, though findings are inconsistent [32,33,34]. CYP2C9 polymorphisms (*2, *3, *13) reduce metabolism to E3174, increasing losartan exposure and diminishing therapeutic effect [13,35,36,37,38]. The frequency of these alleles varies by population [39,40,41].

Physiologically based pharmacokinetic/pharmacodynamic (PBPK/PD) models integrate ADME processes (absorption, distribution, metabolism, and elimination) with drug effects using differential equations [42]. They allow simulation of dosing regimens, organ dysfunction, and genetic variability on drug behavior. In this work, a PBPK/PD model of losartan was developed to investigate dose-dependent PK/PD effects, the influence of hepatic and renal impairment, and the impact of ABCB1 and CYP2C9 variants. The aim is to clarify sources of variability in losartan response and support individualized therapy.

## 2. Materials and Methods

### 2.1. Systematic Literature Research

**Figure 1:**
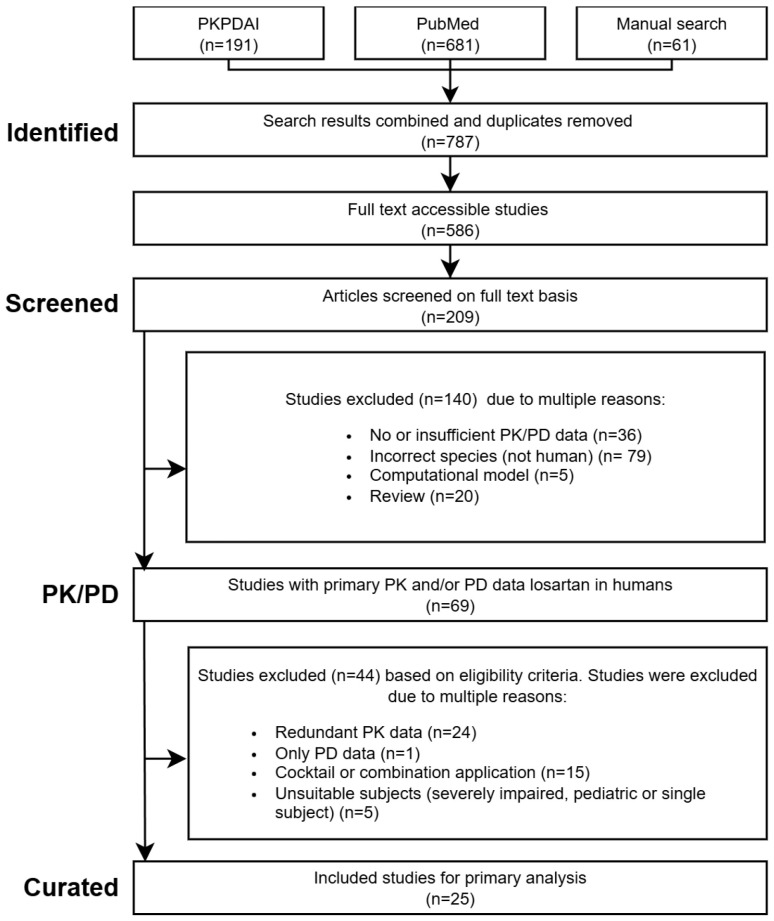
PRISMA flow diagram of the literature search and study selection process. Initially, through the search in PKPDAI, PubMed, and manual search, 787 studies were identified. After removing non-eligible articles, 25 studies were left and curated. PK—pharmacokinetics, PD—pharmacodynamics.

A systematic literature search was performed in PubMed and PKPDAI [43] on 27 August 2024 using the terms losartan AND pharmacokinetics. Eligible studies included human clinical trials with PK/PD data in healthy volunteers, hepatic/renal impairment, or CYP2C9 genotypes. Excluded were animal studies, pediatric/single-patient reports, reviews, computational models, cocktail or combination designs, and PD-only studies. Where multiple studies reported comparable data, representative studies were selected. Studies were excluded if they contained redundant data, such as identical single oral dose study designs (*n* = 44 excluded studies), or if they only reported pharmacodynamic (PD) data without relevant pharmacokinetic (PK) measurements (*n* = 1 excluded study). Additional in vitro reports were included to derive kinetic parameters, but not as studies for primary analysis. An overview of the workflow is provided in Figure 1.

### 2.2. Data Curation

Relevant study data were curated and uploaded to the open pharmacokinetics database PK-DB [44]. Extracted metadata included group and individual characteristics (age, sex, genotype, comorbidities), interventions (dose, route, regimen), and outcomes (concentration–time profiles of losartan and metabolites, RAAS biomarkers, blood pressure, heart rate). Digitization of graphical data was performed using WebPlotDigitizer [45]. Data were organized according to PK-DB standards into groups, individuals, interventions, and time courses, providing the heterogeneous dataset used for modeling and validation.

### 2.3. Computational Model Development

A physiologically based pharmacokinetic/pharmacodynamic (PBPK/PD) model was built in the Systems Biology Markup Language (SBML) [46,47] using sbmlutils [48], visualized using cysbml [49], simulated with sbmlsim [50] based on libroadrunner [51,52], and shared under CC-BY 4.0 at Zenodo (v0.8.0) [53]. The model consists of submodels for intestine, liver, kidney, and RAAS, connected by systemic circulation.

Hepatic impairment was implemented as progressive cirrhosis [54,55], aligned with Child–Pugh classes [56,57]. Renal impairment was modeled as reduced clearance based on glomerular filtration rate following KDIGO guidelines [58,59]. CYP2C9 genetic variability was incorporated using allele-specific activity scaling [60,61,62], and ABCB1 activity was adjusted according to published polymorphism data [63,64].

Fractional organ volumes and blood flows were taken from literature sources [65]. The fractional compartment volumes were set to $FV_{\mathrm{gu}}=1.71\%$ for the gut, $FV_{\mathrm{ki}}=0.44\%$ for the kidneys, $FV_{\mathrm{li}}=2.10\%$ for the liver, and $FV_{\mathrm{lu}}=0.76\%$ for the lungs. Fractional blood flows were defined as $FQ_{\mathrm{gu}}=18.00\%$ for the gut, $FQ_{\mathrm{ki}}=19.00\%$ for the kidneys, $FQ_{h}=21.50\%$ for the hepatic venous outflow, and $FQ_{\mathrm{lu}}=100\%$ for the lungs. Absolute organ volumes and blood flows were calculated by scaling the corresponding fractional values with body weight.

Tissue-to-plasma partition coefficients were assumed to be identical across all tissues for losartan, with a fixed value of $Kp^{\mathrm{LOS}}=3.262$. No tissue partitioning was assumed for the metabolites E3174 and L158. Transport processes of losartan, E3174 and L158 in the liver and kidneys were modeled explicitly.

Multiple-dose regimens were implemented by stepwise numerical integration between dosing intervals, with dosing events applied according to the study-specific protocols. Oral and intravenous doses were specified using the parameters PODOSE_los_ and IVDOSE_los_, respectively. Simulation time horizons and post-dose sampling windows were selected to match the corresponding clinical study designs.

All simulations were performed deterministically using the optimized parameter set representing the typical (mean) individual. Inter-individual or between-subject variability was not included, as the objective was to evaluate typical pharmacokinetic and pharmacodynamic behavior across studies rather than to perform population-based variability analyses. The simulation descriptions of all studies are reported in Supplementary Materials, Section S3, with results of the individual studies provided in Supplementary Materials, Section S4.

The Physiome Journal [66] has demonstrated the reproducibility, reusability, and discoverability of the mathematical model and computational simulations.

### 2.4. Parameter Optimization

Model parameters were estimated using a local optimization approach, and the resulting optimal parameter set was applied consistently across all subsequent simulations without further study–specific refitting. The cost function, defined as a function of the parameter vector $\vec{p}$, minimized the sum of squared, weighted residuals $r_{i,k}$ across all time courses *k* and data points *i*. Time courses were weighted by the number of participants in each study $n_{k}$, and individual time points were weighted by the inverse of the associated measurement uncertainty, represented by the standard deviation $\sigma_{i,k}$. This resulted in weights $w_{i,k}=n_{k}/\sigma_{i,k}$.

$$F\left(\vec{p}\right)=0.5\sum_{i,k}{\left(w_{i,k}\;\cdot \;r_{i,k}\left(\vec{p}\right)\right)}^{2},$$

The weighting by uncertainty weights data points with smaller uncertainty higher, the weighting by participants’ weights data points depending on the number of subjects.

Multiple optimization runs (n=100) were performed using different initial parameter values. Optimization was conducted sequentially: pharmacokinetic parameters were estimated first (Table S2), followed by pharmacodynamic parameters (Table S3).

Goodness of fit was assessed using the root mean squared error (RMSE) and the Akaike information criterion (AIC). RMSE was calculated from the residuals between model predictions $y_{i}^{\mathrm{sim}}$ and observations $y_{i}^{\mathrm{obs}}$ as

$$\mathrm{MSE}=\frac{1}{N}\sum_{i=1}^{N}{\left(y_{i}^{\mathrm{sim}}-y_{i}^{\mathrm{obs}}\right)}^{2},$$

where *N* denotes the number of observations.

$$\mathrm{RMSE}=\sqrt{MSE},$$

AIC was computed assuming normally distributed residuals as

AIC=Nln(MSE)+2k

where *k* is the number of estimated model parameters.

Goodness-of-fit plots with RMSE and AIC are provided in Supplementary Materials, Section S2.

### 2.5. Pharmacokinetic and Pharmacodynamic Parameters

Pharmacokinetic parameters for losartan, E3174, and L158 were derived using non-compartmental methods. The elimination rate ($k_{el}$) was estimated from log-linear regression of the terminal phase. The area under the concentration–time curve (AUC) was computed by the trapezoidal rule and extrapolation. Apparent clearance (Cl/F) and volume of distribution ($V_{d}/F$) were derived from $Cl/F=k_{el}\;\cdot \;V_{d}$ and $V_{d}/F=D/\left(AUC_{\infty }\;\cdot \;k_{el}\right)$, where *D* is dose. Pharmacodynamic outputs (renin, angiotensin I, aldosterone, blood pressure) were summarized by maximal and minimal values.

### 2.6. Sensitivity Analysis

The influence of model parameters on pharmacokinetic (PK) and pharmacodynamic (PD) outcomes was assessed using sensitivity analysis. A reference simulation corresponding to a single oral dose of 10 mg losartan was used. PK readouts comprised the area under the concentration–time curve (AUC), maximum concentration ($C_{\max}$), half-life, volume of distribution ($V_{d}$), clearance (CL), and elimination rate constant ($k_{\mathrm{el}}$) for losartan, as well as AUC, $C_{\max}$, and half-life for its metabolites E3174 and L158. PD readouts included the maximum concentrations of angiotensin I, angiotensin II, and renin, the minimum aldosterone concentration, and changes in systolic blood pressure (SBP), diastolic blood pressure (DBP), and mean arterial pressure (MAP). Parameters representing physical constants, unit conversion factors, and dosing parameters were excluded from the analysis. Results are reported in Supplementary Materials, Section S5.

#### 2.6.1. Sampling-Based Sensitivity Analysis

To quantify uncertainty in PK and PD outcomes arising from variability in model parameters, a sampling-based uncertainty analysis was performed. Model parameters were sampled uniformly within their predefined bounds using Latin hypercube sampling (LHS). A total of n=1000 simulations were conducted, and the resulting distributions of PK and PD readouts were used to characterize parameter-induced uncertainty.

#### 2.6.2. Local Sensitivity Analysis

Local sensitivities were computed by perturbing each parameter $p_{i}$ individually by ±1% relative to its reference value $p_{i,0}$. Sensitivities were calculated using a symmetric midpoint approximation,

$$S\left(q_{k},p_{i}\right)=\frac{q_{k}\left(p_{i}^{+}\right)-q_{k}\left(p_{i}^{-}\right)}{p_{i}^{+}-p_{i}^{-}},$$

where $p_{i}^{\pm }=p_{i,0}\;\cdot \;\left(1\pm 0.01\right)$. Sensitivities were subsequently normalized to obtain dimensionless measures,

$$S_{\mathrm{norm}}\left(q_{k},p_{i}\right)=\frac{q_{k}\left(p_{i}^{+}\right)-q_{k}\left(p_{i}^{-}\right)}{p_{i}^{+}-p_{i}^{-}}\;\cdot \;\frac{p_{i,0}}{q_{k}\left(p_{i,0}\right)},$$

representing the relative change in the model output per relative change in the parameter. Normalized sensitivities with absolute values below 0.1, as well as parameters without measurable effects on any PK or PD readout, were omitted from the heatmap visualization. For clarity, sensitivity matrices were hierarchically clustered by model parameters using single-linkage clustering.

#### 2.6.3. Global Sensitivity Analysis

Global sensitivity analysis was performed using variance-based Sobol indices [67,68,69], as implemented in SALib [70,71]. First-order ($S_{1}$) and total-effect ($S_{T}$) indices were computed. Parameters with $S_{1}$ and S$S_{T}$ values below 0.05, as well as parameters without measurable effects on any readout, were omitted from the heatmap visualization. Samples were generated using Saltelli’s extension of the Sobol sequence with n=4096 base samples.

## 3. Results

### 3.1. Losartan Database

An open database containing pharmacokinetic and pharmacodynamic data of losartan from 25 clinical studies was curated and used for the primary analysis, covering a range of dosing regimens, physiological conditions and different genotypes (Table 1). This dataset served as the basis for developing the losartan PBPK/PD model. Plotted observables and parameter changes per study simulation are reported in Table S4. Study simulations that are not presented in the primary text are reported in the Supplementary Materials (S4).

### 3.2. Computational Model

**Figure 2:**
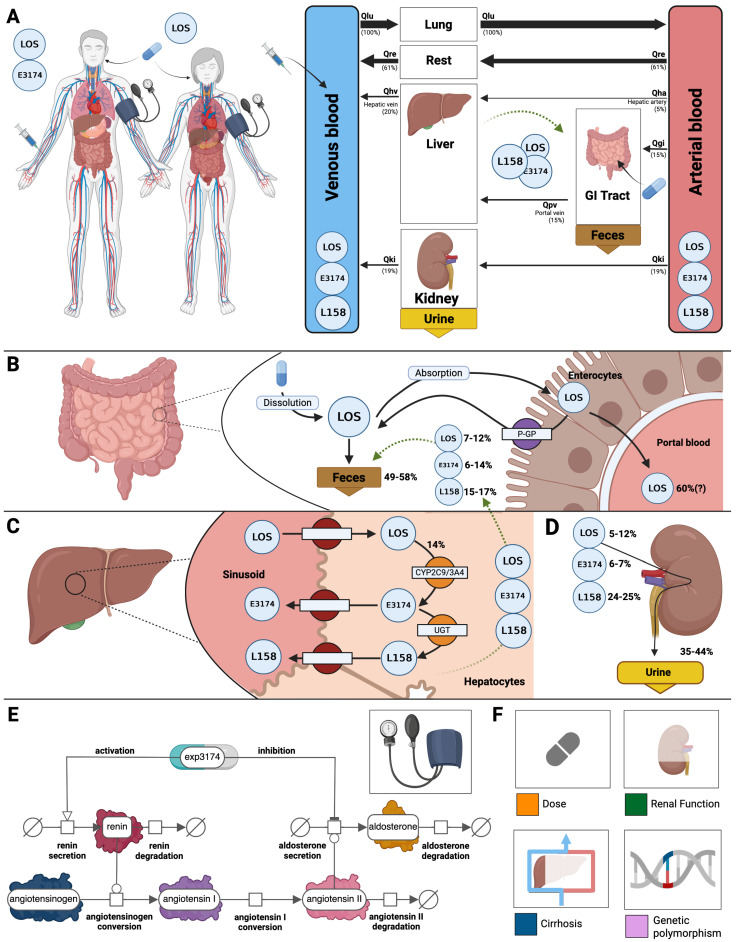
Overview of the physiologically based model of losartan. (**A**) Whole body model showing circulation via the arterial and venous blood, with organs (liver, GI tract, kidney) influencing the pharmacokinetics of losartan (LOS). (**B**) Intestine model illustrating the dissolution and absorption of LOS by enterocytes and the P-glycoprotein-mediated efflux back into the intestine. Approximately 49–58% of the dose is excreted as losartan or metabolites (E3174 and L158). (**C**) Hepatic model depicting the uptake of losartan by hepatocytes and its conversion by cytochrome p450 2C9 and 3A4 (CYP2C9, CYP3A4) to losartan carboxylic acid E3174 (14% of losartan dose) and the following conversion by UDP-glucuronosyltransferase (UGT) to L158. Losartan and its metabolites can also re-enter the intestinal model via enterohepatic circulation (biliary export). (**D**) Renal model showing excretion of losartan, E3174 and L158 via urine, approximately 5–12%, 6–7% and 24–25%, respectively. (**E**) Pharmacodynamic model of E3174 acting on the RAAS. Renin catalyzes the conversion of angiotensinogen to angiotensin I and subsequently to angiotensin II, which stimulates aldosterone secretion. All hormones undergo degradation, while E3174 modulates renin and aldosterone secretion (fe_e3174_). (**F**) Key factors influencing losartan PK and PD profiles accounted for in the model. Illustrations for losartan dosing, hepatic impairment, renal impairment and genetic polymorphisms.

A PBPK/PD model of losartan was created that includes key factors that determine intra- and inter-individual variability (Figure 2). The model includes the organs involved in the pharmacokinetics of losartan (gastrointestinal tract, liver, and kidney), connected through the systemic circulation, as well as the main pharmacodynamic outputs of the RAAS (renin, angiotensin I, aldosterone, systolic and diastolic blood pressure). The pharmacodynamic model focuses on the active metabolite E3174, which mediates the main pharmacodynamic response. The model structure is illustrated in Figure 2E. Renin secretion was modeled as proportional to the drug effect as a function of E3174 concentration. Renin catalyzes the conversion of angiotensinogen to angiotensin I, which is further converted to angiotensin II. Angiotensin II stimulates aldosterone secretion, while all three hormones undergo first-order degradation. The active metabolite E3174 inhibits aldosterone secretion and enhances renin secretion, thereby capturing the dual pharmacodynamic feedback of angiotensin receptor blockade. The resulting set of ordinary differential equations (ODEs) describes time-dependent changes in renin, angiotensin I, angiotensin II, and aldosterone concentrations in plasma. Blood pressure (systolic, diastolic, and mean arterial pressure) was linked to aldosterone concentration through a proportional feedback relationship, using physiological reference values of 120/80 mmHg. This RAAS–E3174 interaction model provides a mechanistic basis for quantifying and predicting losartan’s pharmacodynamic effect under varying physiological and genetic conditions. Specifically, four key factors were included: dose dependency, liver impairment, renal impairment, and genotype variability. Comparison of the introduced model to other published losartan computational models is presented in Table S1.

### 3.3. Dose Dependency

**Figure 3:**
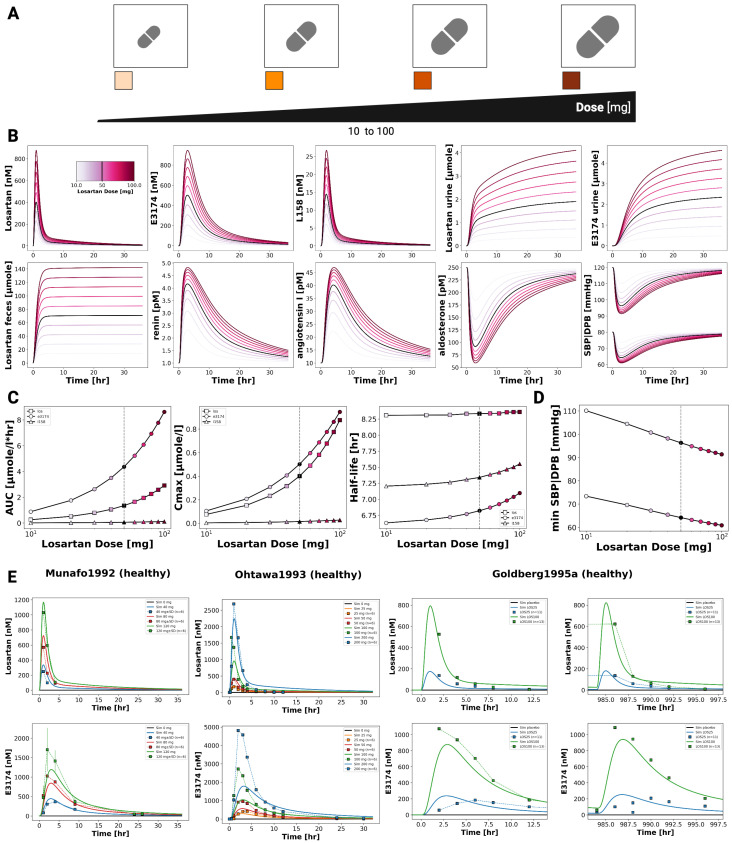

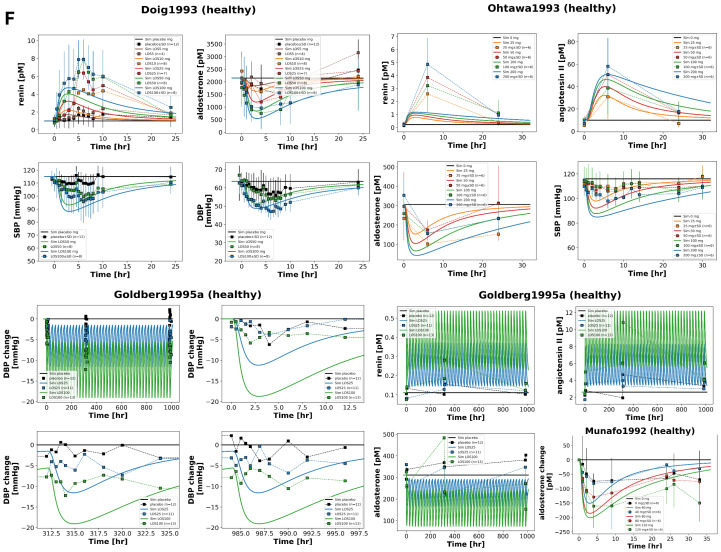
Dose-dependent pharmacokinetics and pharmacodynamics of losartan. (**A**) Illustration of the losartan dose range (10–100 mg) evaluated in the simulations. (**B**) Simulated plasma concentration profiles of losartan, E3174 and L158, urinary excretion profile of losartan and E3174, a fecal excretion profile of losartan, as well as simulated RAAS biomarkers (renin, angiotensin I and aldosterone) and blood pressure responses across doses (10–100 mg). Dose intensity is indicated by line color. (**C**) Dose–response curves for AUC, maximum concentration $C_{\max}$ and half-life. (**D**) Dose dependent maximum or minimum values of systolic blood pressure (SBP) and diastolic blood pressure (DBP). (**E**) Simulated (solid lines) versus observed (symbols) losartan pharmacokinetics. Data from [17,77,83]. (**F**) Simulated (solid lines) versus observed (symbols) losartan pharmacodynamics. Data from [17,75,77,83].

Dose-dependent behavior of the losartan PBPK/PD model was assessed by simulating oral doses of losartan between 10 and 100 mg (Figure 3). The model captures the pharmacokinetics of losartan and its active metabolite E3174 in plasma, urine, and feces, as well as the associated pharmacodynamic responses (aldosterone, renin, angiotensin I, and blood pressure). Simulations reproduced key dose-related trends: increasing exposure with dose, a decreasing E3174/losartan ratio at higher doses, nonlinear increases in AUC and $C_{\max}$ (particularly for E3174), and dose-dependent prolongation of E3174 half-life. On the pharmacodynamic level, higher doses produced stronger suppression of aldosterone and blood pressure, accompanied by compensatory increases in renin and angiotensin I. These patterns are consistent with findings from published clinical studies (Figure 3).

### 3.4. Hepatic Impairment

**Figure 4:**
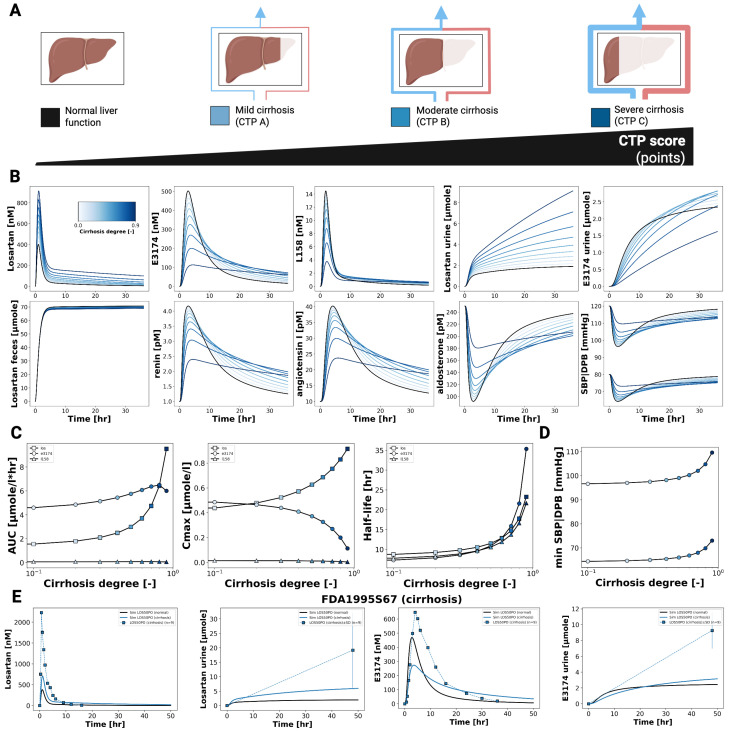
Effect of hepatic impairment on losartan pharmacokinetic and pharmacodynamic profiles. (**A**) Illustration of the level of cirrhosis evaluated in the simulations. (**B**) Simulated plasma concentration profiles of losartan, E3174 and L158, urinary excretion profile of losartan and E3174, a fecal excretion profile of losartan, as well as simulated RAAS biomarkers (renin, angiotensin I and aldosterone) and blood pressure responses across cirrhosis degrees. Cirrhosis degree is indicated by line color. (**C**) Cirrhosis degree-dependent curves for AUC, $C_{\max}$ and half-life. (**D**) Cirrhosis degree-dependent maximum or minimum values of systolic blood pressure SBP and diastolic blood pressure DBP. (**E**) Simulated (solid lines) versus observed (symbols) pharmacodynamic timecourses for different degrees of cirrhosis in FDA1995S67 [19].

Figure 4 shows the impact of hepatic dysfunction on the pharmacokinetics and pharmacodynamics of losartan and E3174. As cirrhosis severity increases, losartan plasma and urine concentrations rise, while E3174 levels and the E3174/losartan ratio decrease. Fecal excretion remains largely unchanged, indicating that hepatic impairment predominantly affects metabolic clearance rather than biliary elimination.

Pharmacokinetic analysis reveals increasing AUC, C_max_, and half-life for both losartan and E3174 with worsening cirrhosis, accompanied by a decline in the elimination rate constant (kel). While losartan accumulation is qualitatively consistent with clinical observations from patients with mild to moderate cirrhosis [8,9], the model underestimates absolute concentrations, particularly for E3174, which shows a delayed but modest increase in plasma levels at higher cirrhosis degrees. This discrepancy likely reflects unaccounted factors such as reduced hepatic blood flow, altered enzyme activity, or additional compensatory mechanisms [28].

Pharmacodynamically, impaired liver function attenuates the formation of E3174 and modifies RAAS regulation. Early renin and angiotensin I responses are reduced, while aldosterone and blood pressure increase initially, with prolonged elevation in more severe cirrhosis. These trends correspond to delayed normalization of hormonal and hemodynamic parameters. Despite sparse PD data and limited control groups, the model reproduces general concentration–time and dose–response patterns, supporting the clinical recommendation of dose adjustment in hepatic impairment. This is particularly relevant for patients with comorbidities such as heart failure, where sensitivity to RAAS inhibition is increased [36,37].

### 3.5. Renal Impairment

**Figure 5:**
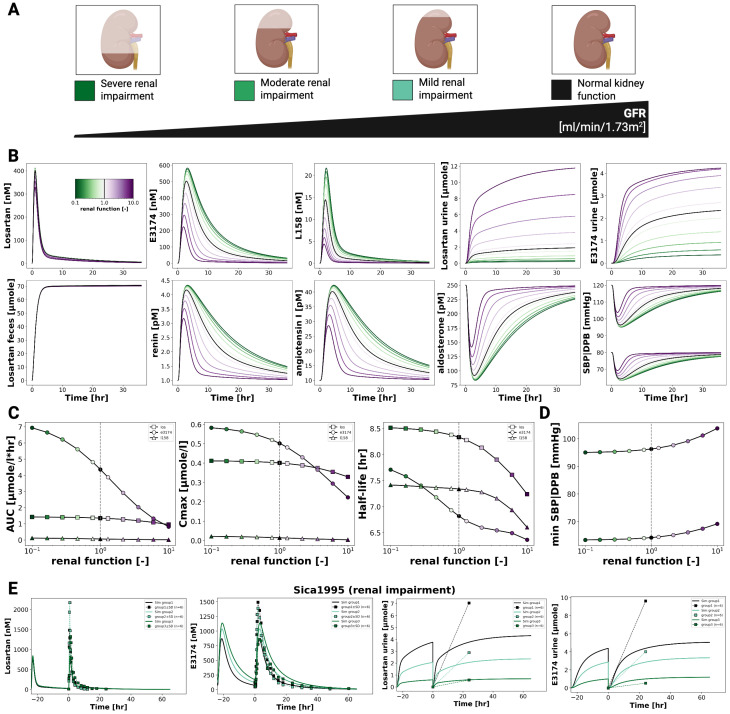
Effect of renal impairment on losartan pharmacokinetics and pharmacodynamic profiles. (**A**) Illustration of the level of renal impairment evaluated in the simulations. (**B**) Simulated plasma concentration profiles of losartan, E3174 and L158, urinary excretion profile of losartan and E3174, a fecal excretion profile of losartan, as well as simulated RAAS biomarkers (renin, angiotensin I and aldosterone) and blood pressure responses across degrees of renal function. Renal function degree is indicated by line color. (**C**) Renal function-dependent curves for AUC, $C_{\max}$ and half-life. (**D**) Renal function-dependent maximum or minimum values of SBP and DBP. (**E**) Simulated (solid lines) versus observed (symbols) pharmacokinetic timecourses for different degrees of renal impairment in Sica1995 [20].

Figure 5 illustrates the effect of declining renal function on the pharmacokinetics and pharmacodynamics of losartan and its active metabolite E3174. As renal function decreases, losartan plasma concentrations remain largely unchanged, whereas E3174 levels increase, accompanied by reduced urinary excretion of both compounds. Fecal excretion of losartan shows a slight compensatory rise, and the E3174/losartan ratio increases with impaired renal clearance.

On the pharmacokinetic level, the model predicts stable AUC and C_max_ values for losartan, while E3174 shows rising AUC, C_max_, and half-life as renal function declines, reflecting reduced clearance. These results align qualitatively with the physiology of renal elimination, though they overestimate metabolite accumulation compared to clinical findings [20,29], where E3174 exposure remained stable across renal function groups. This discrepancy may indicate unaccounted processes such as altered absorption, metabolism, or hepatic clearance of E3174 [9].

Pharmacodynamically, impaired renal function amplifies losartan’s antihypertensive effects. The model predicts stronger suppression of aldosterone and systolic blood pressure, along with compensatory rises in renin and angiotensin I. While direct PD data in renally impaired populations are limited, these predictions are physiologically plausible and extend clinical observations. Nevertheless, the degree of RAAS suppression may be slightly overestimated due to the absence of compensatory mechanisms such as altered hepatic clearance or long-term feedback regulation [19,37,38].

### 3.6. ABCB1 Genotypes

**Figure 6:**
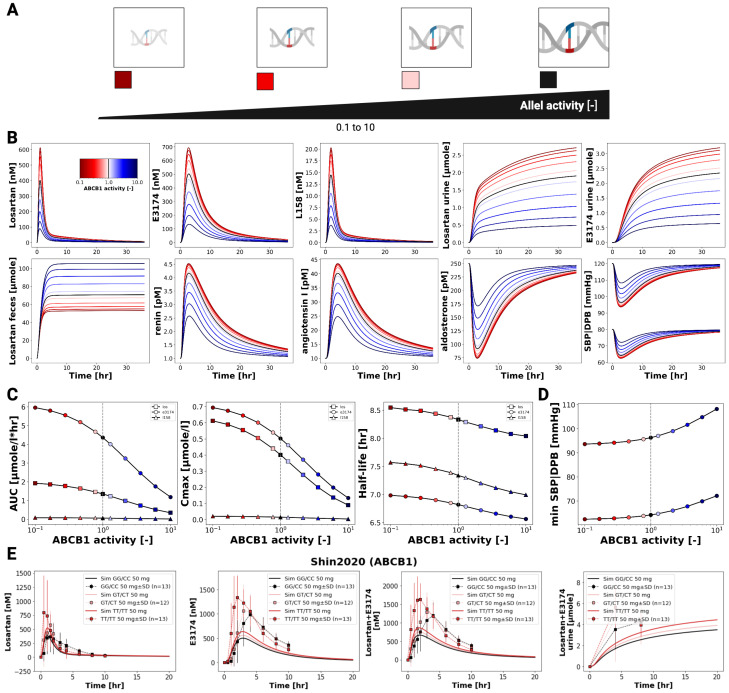
Pharmacokinetics and pharmacodynamics of losartan and metabolites across varying ABCB1 activity levels (**A**) Illustration of the level of CYP2C9 activity evaluated in the simulations. (**B**) Simulated plasma concentrations and excretion profiles of losartan, E3174 and L158, urinary excretion profile of losartan and E3174, a fecal excretion profile of losartan, as well as simulated RAAS biomarkers (renin, angiotensin I and aldosterone) and blood pressure responses across varying ABCB1 activity. Allele function degree is indicated by color. (**C**) ABCB1 activity-dependent curves for AUC, $C_{\max}$ and half-life. (**D**) ABCB1 allele activity-dependent maximum or minimum values of SBP and DBP. (**E**) Simulated (solid lines) versus observed (symbols) pharmacodynamic timecourses for different degrees of ABCB1 activity in Shin2020 [33].

Figure 6 illustrates the influence of ABCB1 (P-glycoprotein) transporter activity on the pharmacokinetics and pharmacodynamics of losartan and E3174. Simulations across varying transporter activity levels, including common diplotypes (GG/CC, GT/CT, TT/TT), show that reduced ABCB1 activity leads to increased plasma and urinary concentrations of both compounds, while fecal excretion of losartan declines due to decreased intestinal and biliary efflux.

Pharmacokinetic analysis indicates modest increases in AUC and C_max_ for losartan and E3174 with lower ABCB1 activity, whereas elimination rate constants (kel) and half-life remain largely unchanged. The plasma and urine E3174/losartan ratio decreases slightly under low transporter activity. These trends are qualitatively consistent with clinical observations [33], although absolute concentrations are slightly underestimated by the model.

Pharmacodynamically, reduced ABCB1 activity slightly enhances RAAS-related effects, including modest increases in renin and angiotensin I and marginal reductions in aldosterone and blood pressure. While these changes are small and unlikely to necessitate dose adjustments based on ABCB1 diplotype alone, the model demonstrates the capacity to predict subtle genotype-dependent differences in drug exposure and pharmacodynamic response. Future refinement could include better representation of renal P-gp expression and its contribution to systemic clearance, as well as additional clinical data on pharmacodynamic outcomes to improve predictive accuracy [33].

### 3.7. CYP2C9 Genotypes

**Figure 7:**
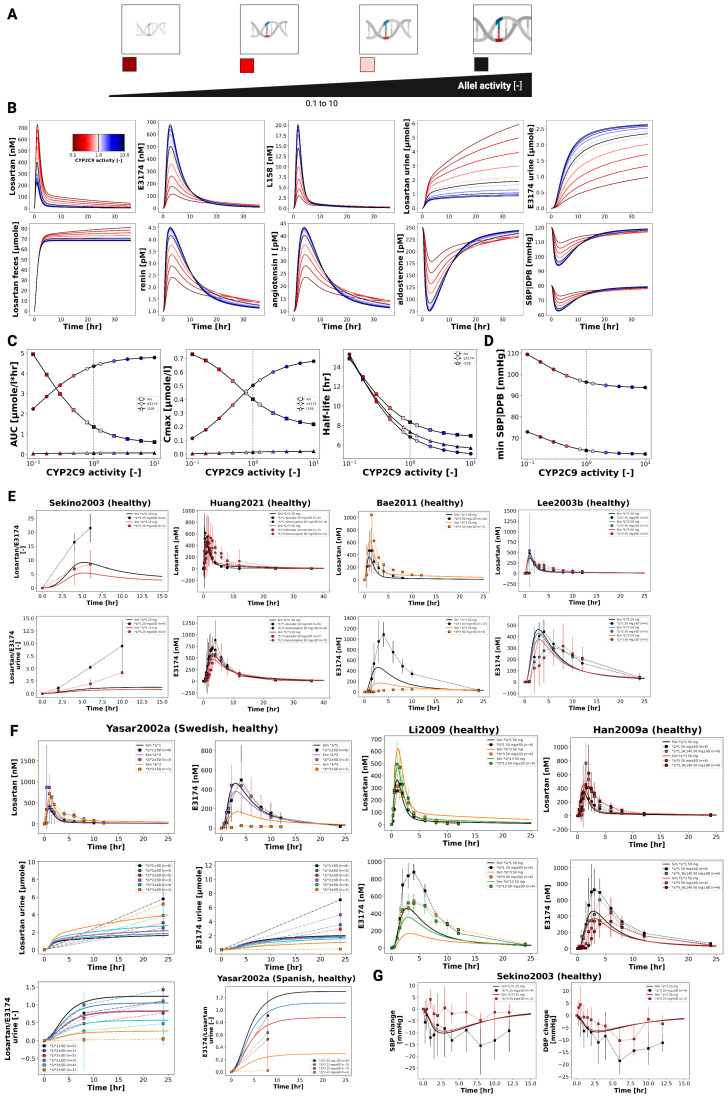
Pharmacokinetics and pharmacodynamics of losartan and metabolites across varying CYP2C9 activity levels. (**A**) Illustration of the level of CYP2C9 activity evaluated in the simulations. (**B**) Simulated plasma concentrations and excretion profiles of losartan, E3174 and L158, urinary excretion profile of losartan and E3174, a fecal excretion profile of losartan, as well as simulated RAAS biomarkers (renin, angiotensin I and aldosterone) and blood pressure responses across varying CYP2C9 activity. Activity degree is indicated by color. (**C**) CYP2C9 activity-dependent curves for AUC, $C_{\max}$, and half-life. (**D**) CYP2C9 allele activity-dependent maximum or minimum values of SBP and DBP. (**E**) Simulated (solid lines) versus observed (symbols) losartan pharmacokinetics. Data from [37,73,79,82]. (**F**) Simulated (solid lines) versus observed (symbols) losartan pharmacokinetics. Data from [36,41,78]. (**G**) Simulated (solid lines) versus observed (symbols) SBP and DBP for different CYP2C9 genotypes [37].

Figure 7 illustrates the impact of CYP2C9 genetic variability on the pharmacokinetics and pharmacodynamics of losartan and its active metabolite E3174. Simulations across a continuous range of CYP2C9 activity, as well as genotype-specific simulations for *1/*1, *1/*2, *1/*3, *1/*13, *2/*2, *2/*3, and *3/*3, demonstrate that reduced enzyme activity leads to impaired metabolic conversion, resulting in higher plasma, urine, and fecal levels of losartan and decreased E3174 exposure.

Pharmacodynamically, lower CYP2C9 activity diminishes RAAS inhibition, with smaller reductions in aldosterone and systolic blood pressure, and attenuated compensatory increases in renin and angiotensin I. AUC and C_max_ for losartan rise with declining enzyme activity, while E3174 exposure decreases; elimination rate constants and half-life remain relatively stable.

These model predictions are consistent with clinical observations, which report 1.6- to 3-fold increases in losartan AUC and significantly reduced E3174 levels in carriers of reduced-function alleles [36,37,38], although absolute concentrations tend to be slightly underestimated (Figure 7).

The use of a continuous CYP2C9 activity scale enables the simulation of nonlinear relationships between enzyme activity and metabolite formation across a spectrum of genotypes. While the CYP2C9 genotype is a major determinant of inter-individual variability, other factors such as age, comorbidities, and concomitant medications may further influence pharmacokinetics and pharmacodynamics. From a clinical perspective, these findings support the potential of genotype-guided dosing for patients with reduced-function alleles, although high variability within genotype groups suggests that genetic testing should be applied selectively, focusing on individuals with inadequate response or elevated risk of adverse effects.

## 4. Discussion

In this study, we developed and evaluated a physiologically based pharmacokinetic/pharmacodynamic (PBPK/PD) model of losartan, informed by a curated database of clinical studies covering diverse dosing regimens, populations, and physiological conditions. The model integrates intestinal absorption, hepatic metabolism via CYP2C9, and fecal and renal excretion, while linking systemic exposure of the active metabolite E3174 to downstream pharmacodynamic effects through a simplified RAAS submodel. This framework enables simulation of losartan and metabolite concentrations in plasma, urine, and feces, as well as prediction of pharmacodynamic endpoints such as renin, angiotensin I, aldosterone, and systolic and diastolic blood pressure. By reproducing key clinical observations, the model demonstrates its potential to provide mechanistic insights into inter-individual variability and support personalized dosing strategies.

Our dose dependency simulations reproduced the nonlinear pharmacokinetics of losartan and E3174, demonstrating a decreasing metabolite-to-parent ratio at higher doses due to saturable CYP2C9 metabolism [17,77,83]. Consistent with clinical findings, increasing doses resulted in stronger and more sustained suppression of aldosterone and systolic blood pressure, accompanied by compensatory increases in renin and angiotensin I [17,75,77]. This supports the model’s ability to capture both nonlinear PK behavior and dose-dependent RAAS responses.

In hepatic impairment, the model predicted elevated losartan plasma concentrations, reduced clearance, and attenuated metabolite formation, reflecting impaired CYP2C9 metabolism and hepatic blood flow. While the model underestimated the reported accumulation of E3174 in cirrhosis in comparison to clinical findings [19], it nonetheless confirmed the need for dose reduction in patients with liver dysfunction, particularly in comorbid conditions such as heart failure, where sensitivity to RAAS inhibition is heightened.

Renal impairment simulations predicted reduced clearance of both losartan and E3174, leading to prolonged systemic exposure. While these results are qualitatively consistent with PK data, the model overestimated E3174 accumulation compared to reported clinical findings. Importantly, in the absence of PD data from impaired populations, the model allowed prediction of enhanced RAAS suppression and blood pressure reduction—although these effects may be slightly exaggerated due to the omission of compensatory mechanisms.

Genetic variability was also explored. For CYP2C9, the model captured the pronounced impact of reduced-function alleles on E3174 formation and downstream pharmacodynamic effects, reproducing the diminished blood pressure reduction observed clinically. The use of a continuous enzyme activity scale enabled simulation across a spectrum of genotypic variability. In contrast, ABCB1 (P-gp) variability had only minor effects on systemic exposure and blood pressure, consistent with limited clinical data, although the model suggests possible contributions via altered intestinal efflux and renal secretion. These findings emphasize the potential of PBPK/PD modeling to disentangle the contributions of genetic variability to losartan response.

The mechanistic insights provided by this PBPK/PD model offer a direct pathway to actionable clinical recommendations, particularly through integration with Electronic Health Records (EHRs). For example, our model-based approach for CYP2C9 genotype-guided dosing could be implemented as a Clinical Decision Support tool within the EHR. When a patient’s genotype data (already in the record from pharmacogenomic testing) and clinical covariates (e.g., age, liver function score) are entered, the PBPK/PD model could run a rapid simulation to predict their individual E3174 exposure and blood pressure response. This simulation output could then be translated into a model-informed starting dose recommendation that is automatically flagged for the prescribing physician, thereby moving beyond static dosing guidelines to true precision medicine.

The curated database was essential for calibration and validation of the model, but it also highlights important limitations. While losartan pharmacokinetics are well characterized, data on RAAS dynamics remain limited, particularly with respect to circadian variability and long-term feedback regulation. Because RAAS components exhibit pronounced time-of-day oscillations, the absence of circadian data may influence the accuracy of long-term or steady-state predictions, especially for endpoints such as renin or aldosterone that are highly rhythm-dependent. Incorporating such rhythms could improve prediction and support chronopharmacological dosing evaluations. Excretion data, especially for fecal elimination and the secondary metabolite L158, were sparse, and pharmacodynamic datasets showed high variability across studies. These gaps restrict the predictive accuracy of the model under complex physiological conditions but do not preclude its use in capturing system-level dose–exposure–response relationships.

The model links systemic exposure of the active metabolite E3174 to its pharmacodynamic effects via a simplified RAAS submodel. RAAS regulation is inherently complex and influenced by multiple factors, many of which were outside the scope of this model. Physiologically, angiotensin II (Ang II) regulates extracellular volume and blood pressure primarily through five key physiological actions. These actions include: vasoconstriction by contraction of the vascular smooth muscle in the arterioles; stimulation of aldosterone secretion from the adrenal cortex to promote sodium and water retention; increased renal sodium reabsorption in the proximal convoluted tubule (PCT) via the Na-H antiporter; enhanced sympathetic nervous system outflow; and the release of vasopressin (ADH) from the hypothalamus [12]. Our model focuses on the aldosterone-mediated effect on blood pressure. More precisely, blood pressure changes were modeled as linearly proportional to aldosterone deviations from baseline. Although simplified, this structure allows quantitative assessment of the relationship between E3174 plasma levels and RAAS-mediated changes in blood pressure. The model also lacks feedback regulation and circadian rhythmicity, two critical components of RAAS physiology. As a result, it can simulate initial pharmacodynamic responses but not long-term homeostatic adaptations.

A limitation of the present work is that parameter uncertainty was not explicitly quantified using stochastic simulations or Bayesian inference approaches. While the conducted local, global, and sampling-based sensitivity analyses provide insight into the relative impact of individual parameters on pharmacokinetic and pharmacodynamic outcomes, they do not capture the full propagation of physiological and parameter variability to predicted clinical responses. Future extensions of this digital twin framework will therefore focus on integrating Monte Carlo–based uncertainty propagation and Bayesian parameter estimation to systematically quantify uncertainty, improve model interpretability, and enhance the clinical robustness of personalized blood pressure predictions.

Looking forward, several opportunities for refinement exist. Incorporating circadian rhythm, sodium and water balance, and autonomic feedback would enhance physiological fidelity of the RAAS submodel. Future model extensions could also integrate additional sources of variability, such as age, comorbidities, or transporter–enzyme interactions, to support population-level simulations. Another promising direction is to couple the mechanistic PBPK/PD model with population-based modeling frameworks or machine-learning approaches. Hybrid models could leverage large observational datasets to estimate variability distributions, refine parameter priors, or emulate complex nonlinear dynamics that are difficult to represent mechanistically. Such integration would broaden the applicability of the model to population-level prediction, risk stratification, and adaptive dosing. Ultimately, the losartan PBPK/PD framework provides a mechanistically grounded platform for exploring inter-individual variability and optimizing dosing strategies, with potential applications in precision medicine and clinical decision support.

## 5. Conclusions

In this study, a comprehensive physiologically based pharmacokinetic/pharmacodynamic (PBPK/PD) model of losartan and its active metabolite, E3174, was developed and validated using an extensive clinical database. The model successfully reproduced key pharmacokinetic and pharmacodynamic behaviors, including dose-dependent RAAS inhibition and nonlinear metabolite formation resulting from saturable CYP2C9 metabolism, and the pronounced impact of CYP2C9 genetic variability on blood pressure response. Simulations captured the attenuated pharmacodynamic response under hepatic impairment and predicted enhanced RAAS suppression in renal dysfunction. Furthermore, the model quantified the pronounced impact of CYP2C9 genetic variability on metabolite exposure and blood pressure effects, while ABCB1 activity contributed only minor modulation of systemic exposure.

Despite certain limitations in clinical data and model scope, the framework provides valuable mechanistic insight into inter-individual variability in losartan response. It highlights the potential of systems pharmacology modeling to support dose optimization across physiological and genetic conditions. Together, the losartan database and PBPK/PD model establish a solid foundation for future integration into digital twin platforms and personalized therapy design.

Moving forward, development could focus on two key areas to enhance clinical relevance. First, a structural refinement of the model by incorporating dynamic variables such as liver blood flow and glomerular filtration rate (GFR) would improve the predictive accuracy of E3174 exposure in organ-impaired populations. Second, and critically, a prospective validation of the model against new, independent clinical data to formally verify its predictive performance could be conducted. Together, the losartan database and refined PBPK/PD model could establish a reliable platform for future integration into clinical decision support systems, digital twin platforms, and personalized anti-hypertensive therapy design.

## Figures and Tables

**Table 1:** Summary of studies for modeling. Overview of study identifiers, PK-DB IDs, administered substance and administration route, dosing regimens, doses [mg], and subject characteristics, including health status, renal functional impairment (RFI), hepatic functional impairment (HFI), and the studied genotypes (CYP2C9, ABCB1).

Study	PK-DB	Substance	Route	Dosing	Dose [mg]	Healthy	RFI	HFI	CYP2C9	ABCB1
Azizi1999 [72]	PKDB00997	losartan potassium	po	single	50	✔			✔	
Bae2011 [73]	PKDB00895	losartan potassium	po	single	50	✔			✔	
Donzelli2014 [74]	PKDB00953	losartan	po	single	12.5	✔			✔	
Doig1993 [75]	PKDB00976	losartan	po	single	5, 10, 25, 50, 100	✔			✔	
FDA1995S60 [19]	PKDB00965	C14 losartan, e3174	po/iv, iv	single	100/30, 20	✔			✔	
FDA1995S67 [19]	PKDB00966	losartan, e3174	po/iv, iv	single	50/10, 10			✔		
Fischer2002 [38]	PKDB00894	losartan	po	multi	50	✔			✔	
Goldberg1995 [76]	PKDB00975	losartan	po	single	100	✔			✔	
Goldberg1995a [77]	PKDB00974	losartan potassium	po	single	25, 100	✔			✔	
Han2009a [78]	PKDB00909	losartan potassium	po	single	50	✔			✔	
Huang2021 [79]	PKDB00919	losartan potassium	po	single	50	✔			✔	
Kim2016 [80]	PKDB00896	losartan	po	single	50	✔				
Kobayashi2008 [81]	PKDB00920	losartan potassium	po	single	25	✔				
Lee2003b [82]	PKDB00899	losartan potassium	po	single	50	✔			✔	
Li2009 [41]	PKDB00912	losartan	po	single	50	✔			✔	
Lo1995 [13]	PKDB00922	losartan potassium, e3174	po/iv	single	50, 100/20, 30	✔				
Munafo1992 [83]	PKDB00921	losartan	po	single	40, 80, 120	✔				
Oh2012 [84]	PKDB00054	losartan	po	single	2	✔				
Ohtawa1993 [17]	PKDB00911	losartan	po	single, multi	25, 50, 100, 200	✔				
Puris2019 [85]	PKDB00642	losartan potassium	po	single	12.5					
Sekino2003 [37]	PKDB00961	losartan	po	single	25	✔			✔	
Shin2020 [33]	PKDB00898	losartan potassium	po	single	50	✔				✔
Sica1995 [20]	PKDB00910	losartan	po	multi	100		✔			
Tanaka2014 [86]	PKDB00136	losartan	po	single	50	✔				
Yasar2002a [36]	PKDB00897	losartan	po	single	50	✔			✔	

## Data Availability

All curated pharmacokinetic data are publicly available in the PK-DB database (https://pk-db.com). The model and all associated materials (simulation scripts, parameters, and documentation) are publicly available in SBML format under a CC-BY 4.0 license at https://github.com/matthiaskoenig/losartan-model (accessed on 12 January 2026) [53].

## Supplementary Materials

The following supporting information can be downloaded at https://www.mdpi.com/article/10.3390/pharmaceutics18020262/s1, Table S1: Summary of published computational models for losartan, Table S2: Optimized parameters for the losartan pharmacokinetic model, Table S3: Optimized parameters for the losartan pharmacodynamic model, Table S4: Goodness of fit metrics, Table S5: Plotted observables and parameter changes per study simulation, Table S6: Plotted observables and parameter changes per simulation experiment and scan, Table S7. Model parameters. Table S8. Sampling-based parameter sensitivity metrics. Figure S1: Optimization performance, Figure S2: Simulation Azizi1999, Figure S3: Simulation Donzelli2014, Figure S4: Simulation FDA1995S60, Figure S5: Simulation FDA1995S60, Figure S6: Simulation FDA1995S60 Figure S7: Simulation Fischer2002, Figure S8: Simulation Goldberg1995, Figure S9: Simulation Kim2016, Figure S10: Simulation Kobayashi2008, Figure S11: Simulation Lo1995, Figure S12: Simulation Lo1995, Figure S13: Simulation Lo1995, Figure S14: Simulation Oh2012, Figure S15: Simulation Puris2019, Figure S16: Simulation Tanaka2014, Figure S17: Sampling-based parameter sensitivity, Figure S18: Normalized local parameter sensitivity, Figure S19: Global parameter sensitivity (Sobol S1), Figure S20: Global parameter sensitivity (Sobol ST).
