# Supplementary material for: A Digital Twin of the Angiotensin II Receptor Blocker Losartan: Physiologically Based Modeling of Blood Pressure Regulation
Source: Pharmaceutics. 2026 Feb 19;18(2):262. doi: 10.3390/pharmaceutics18020262 (PMC12944407; doi:10.3390/pharmaceutics18020262)
Supplement: Supplementary file 1 [file pharmaceutics-18-00262-s001.zip › pharmaceutics-3972571-supplementary.pdf]

Supplementary Materials: A Digital Twin of the Angiotensin II Receptor Blocker Losartan: Physiologically Based Modeling of Blood Pressure Regulation

Ennie TENSIL, Mariia MYSHKINA, and Matthias KÖNIG

Contents

S1. Published Losartan Models (Tab. S1) **2**

S2. Parameter Optimization **3**
- S2.1. Optimal Parameters (Tab. S2, S3) 3
- S2.2. Parameter Optimization Results (Fig. S1, Tab. S4) 4

S3. Simulations Description **4**

S4. Simulations **7**
- S4.1. Azizi1999 (Fig. S2) 7
- S4.2. Donzelli2014 (Fig. S3) 8
- S4.3. FDA1995S60 (Fig. S4, S5, S6) 8
- S4.4. Fischer2002 (Fig. S7) 11
- S4.5. Goldberg1995 (Fig. S8) 11
- S4.6. Kim2016 (Fig. S9) 12
- S4.7. Kobayashi2008 (Fig. S10) 12
- S4.8. Lo1995 (Fig. S11, S12, S13) 13
- S4.9. Oh2012 (Fig. S14) 14
- S4.10. Puris2019 (Fig. S15) 15
- S4.11. Tanaka2014 (Fig. S16) 16

S5. Model Parameters (Tab. S7) **17**

S6. Parameter Sensitivity Analysis **18**
- S6.1. Sampling Sensitivity Analysis (Fig. S17, Tab. S8) 18
- S6.2. Local Sensitivity Analysis (Fig. S18) 19
- S6.3. Global Sensitivity Analysis (Fig. S19, S20) 20

S7. References **21**

S1. Published Losartan Models (Tab. S1)

Table S1. Summary of published computational models for losartan. Overview of published computational models including model type, software/platform, reproducibility criteria (open software, open model, open code, open data, open license, reproducibility, FAIR, long-term storage), resources, clinical data sources, and model scope.

Study	PubMed ID	Model Type	Platform/Software	Open Software	Open Model	Open Code	Open Data	Open License	Reproducibility	FAIR	Long-term Storage	Resources	Studies	Clinical Data Used	Scope
Babaev2025 [1]	40810118	Mechanistic PK/PD	BioUML, SBML, SBGN	Yes	Yes	Yes	Yes	Yes	Yes	Yes	Yes	Biomodels, GitHub, GitLab	3	3 clinical studies; referenced & digitized literature data	CYP2C9 genotype dependent losartan PK, active metabolite formation, blood pressure response in virtual hypertensive patients
Babaev2025b [2]	40733143	Mechanistic PK/PD	BioUML, SBML, SBGN	Yes	Yes	Yes	Yes	Yes	Yes	Yes	Yes	Biomodels, GitHub, GitLab	4	4 clinical studies; referenced & digitized literature data	CYP2C9 and ABCB1 genotype dependent losartan PK, transporter effects, BP response in virtual hypertensive patients
Gardiner2011 [3]	21778353	Mechanistic PBPK	Berkeley Madonna, WinNonlin	No	No	No	No	No	No	No	No	-	Multiple	Generated de novo data (not publicly available)	Hepatic uptake transport, impact on organic anion PK
Karatza2020 [4]	31514255	Mechanistic PK	Monolix Suite	No	No	No	No	No	No	No	No	-	1	Single crossover bioequivalence study (not publicly available)	Gastric emptying driven losartan and EXP-3174 PK, parent-metabolite absorption dynamics
Karatza2021 [5]	33768449	Mechanistic PK	Monolix, Matlab, R	No	No	No	No	No	No	No	No	-	1	Referenced clinical bioequivalence study data (confidential)	Gastric emptying driven losartan PK, multiple peak phenomenon, PCA of PK parameters
Nguyen2017 [6]	28412400	Mechanistic PBPK	GastroPlus, Simcyp	No	No	No	No	No	No	No	No	-	Multiple	Referenced & digitized literature clinical PK data (not shared)	Losartan and EXP-3174 PBPK, transporter mediated hepatic disposition, metabolite exposure prediction
Ramusovic2012 [7]	23143654	Mechanistic PK/PD	Mobi, PK-Sim	No	No	No	No	No	No	No	No	-	Multiple	Referenced & digitized literature clinical data (not shared)	RAAS biomarkers, multi-drug interaction
Tensil2025	-	PBPK/PD	SBML, Python	Yes	Yes	Yes	Yes	Yes	Yes	Yes	Yes	GitHub, Zenodo	25	25 clinical studies; digitized literature data	Physiologically based PK/PD model of losartan integrating systemic exposure, metabolite formation, and RAAS-mediated blood pressure regulation across dose, organ impairment, and genetic variability

S2. Parameter Optimization

S2.1. Optimal Parameters (Tab. S2, S3)

Table S2. Optimized parameters for the losartan pharmacokinetic model. Lower (LB) and upper (UB) bounds applied during parameter optimization.

Parameter name	Description	Value	Unit	LB	UB
`ftissue_los`	Tissue blood flow rate for LOS	0.145	l/min	0.01	10
`Kp_los`	Tissue-to-plasma partition coefficient of LOS	3.262	-	1	200
`GU__LOSABS_k`	Absorption rate of LOS in the gastrointestinal tract (GI) tract	0.036	1/min	1×10^{-4}	1
`GU__f_LOSEFL_k`	Rate for LOS efflux (P-glycoprotein) relative to absorption	1.020	-	0.1	10
`GU__METEXC_k`	Rate of metabolite feces excretion	2.696×10^{-5}	l/min	1×10^{-5}	0.1
`LI__E3174EX_k`	Rate E3174 export from hepatocytes to systemic circulation	0.011	1/min	1×10^{-3}	10
`LI__LOS2E3174_Vmax`	Liver metabolism of LOS to E3174	7.251×10^{-4}	mmol/min/l	1×10^{-5}	100
`LI__E3174L158_k`	Liver metabolism of E3174 to L158	1.134×10^{-3}	1/min	1×10^{-5}	10
`LI__MBIEX_k`	Rate for LOS, E3174 and L158 export in bile to GI	0.066	1/min	1×10^{-5}	1
`KI__LOSEX_k`	Renal excretion rate of LOS	0.077	1/min	1×10^{-4}	1
`KI__E3174EX_k`	Renal excretion rate of E3174	0.029	1/min	1×10^{-4}	1
`KI__L158EX_k`	Renal excretion rate of L158	0.289	1/min	1×10^{-4}	1

Table S3. Optimized parameters for the losartan pharmacodynamic model. Lower (LB) and upper (UB) bounds applied during parameter optimization.

Parameter name	Description	Value	Unit	LB	UB
`ANGGEN2ANG1_k`	Rate of angiotensinogen to angiotensin I conversion	0.100	l/min	0.01	1×10^{6}
`E50_e3174`	Half-maximum effect concentration E3174	2.911×10^{-4}	mM	5×10^{-7}	0.05
`ALDSEC_k`	Rate of aldosterone secretion	1.011	mmole/min	5×10^{-6}	1
`BP_ald_fe`	Effect of aldosterone on blood pressure	0.312	-	0.1	0.6

S2.2. Parameter Optimization Results (Fig. S1, Tab. S4)

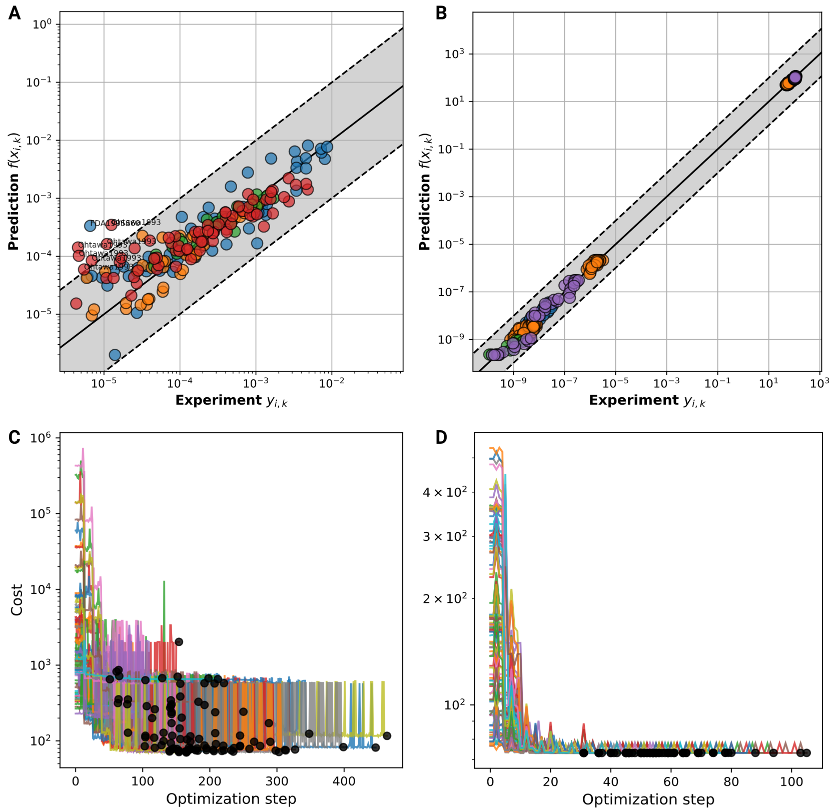

Figure S1. Optimization Performance. Goodness-of-fit (predicted vs. observed intravenous data) and error convergence (cost vs. optimization step) for pharmacokinetic (**A**, **C**) and pharmacodynamic (**B**, **D**) parameter sets.

Table S4. Goodness of fit metrics. N: number of data points, k: number of parameters, MSE: mean squared error, RMSE: root mean squared error, AIC: Akaike Information Criterion.

uid	name	N	k	MSE	RMSE	AIC
20250708_183921__4fba0	LOSARTAN_LSQ_PK	225	12	70.15	8.38	980.39
20250711_231400__8d0b3	LOSARTAN_LSQ_PD	460	4	73.10	8.55	1982.22

S3. Simulations Description

Table S5. Plotted observables and parameter changes per study simulation. Square brackets around SBML species ids indicate concentrations (amount/volume units). Square brackets enclosing numerical values indicate parameter ranges, whereas curly brackets indicate sets of discrete choices.

StudyID	Plotted	Changes
Azizi1999 [8]	[Cve_los], [Cve_e3174], [ren], [ang1], [ang2], MAP	PODOSE_los ∈ {0, 50} mg<br>ren_ref, [ren] = 58.5 pg/ml<br>ang1_ref, [ang1] = 11.8 pg/ml<br>ang2_ref, [ang2] = 7.2 pg/ml<br>SBP_ref = 120 mmHg<br>DBP_ref = 70.5 mmHg
Bae2011 [9]	[Cve_los], [Cve_e3174]	PODOSE_los = 50 mg<br>LI__f_cyp2c9 ∈ {1.0, 0.17}

Table S5. Plotted observables and parameter changes per study simulation (continued). Square brackets around SBML species ids indicate concentrations (amount/volume units). Square brackets enclosing numerical values indicate parameter ranges, whereas curly brackets indicate sets of discrete choices.

StudyID	Plotted (sid)	Changes
Doig1993 [10]	[ren], [ald], ald_change, ald_ratio, SPB, DBP	PODOSE_los ∈ {0, 5, 10, 25, 50, 100} mg<br>BW = 76.5 kg<br>ren_ref, [ren] = 44.8 pg/ml<br>ald_ref, [ald] = 774.12 pg/ml<br>SBP_ref = 115 mmHg<br>DBP_ref = 63.5 mmHg
Donzelli2014 [11]	[Cve_los], [Cve_e3174]	PODOSE_los = 12.5 mg
FDA1995S60 [12]	[Cve_los], Aurine_los, Afeces_los, [Cve_e3174], Aurine_e3174, Afeces_e3174, [Cve_l158], Aurine_l158, Afeces_l158, [Cve_total], Aurine_total, Afeces_total	PODOSE_los = 100 mg<br>IVDOSE_los = 30 mg<br>IVDOSE_e3174 = 20 mg<br>BW = 78.6 kg
FDA1995S67 [13]	[Cve_los], Aurine_los, [Cve_e3174], Aurine_e3174	PODOSE_los = 50 mg<br>IVDOSE_los = 10 mg<br>IVDOSE_e3174 = 10 mg<br>BW = 82.3 kg<br>f_cirrhosis ∈ {0.0, 0.67}
Fischer2002 [14]	[Cve_los], Aurine_los, [Cve_e3174], Aurine_e3174	PODOSE_los = 50 mg<br>BW = 72 kg
Goldberg1995 [15]	[Cve_los], [Cve_e3174], [ren]	PODOSE_los ∈ {0, 50} mg<br>ren_ref, [ren] = 10.5 pg/ml
Goldberg1995a [16]	[Cve_los], [Cve_e3174], [ren], [ang2], [ald], DBP_change	PODOSE_los ∈ {0, 25, 100} mg<br>ren_ref, [ren] = 5.02 pg/ml<br>ang2_ref, [ang2] = 2.71 pg/ml<br>ald_ref, [ald] = 11.2 ng/dl
Han2009a [17]	[Cve_los], [Cve_e3174]	PODOSE_los = 50 mg<br>LI__f_cyp2c9 ∈ {1.0, 0.585}
Huang2021 [18]	[Cve_los], [Cve_e3174]	PODOSE_los = 50 mg<br>BW ∈ {54, 52} kg<br>LI__f_cyp2c9 ∈ {1.0, 0.585}
Kim2016 [19]	[Cve_los], [Cve_e3174]	PODOSE_los = 25 mg<br>BW = 62.3 kg
Kobayashi2008 [20]	[Cve_los], [Cve_e3174]	PODOSE_los = 50 mg
Lee2003b [21]	[Cve_los], [Cve_e3174]	PODOSE_los = 50 mg<br>LI__f_cyp2c9 ∈ {1.0, 0.8, 0.585}
Li2009 [22]	[Cve_los], [Cve_e3174]	PODOSE_los = 50 mg<br>LI__f_cyp2c9 ∈ {1.0, 0.17, 0.525}
Lo1995 [23]	[Cve_los], Aurine_los, [Cve_e3174], Aurine_e3174	PODOSE_los ∈ {50, 100} mg<br>Ri_los ∈ {0.0, 1.0, 1.5} mg/min<br>Ri_e3174 ∈ {0, 1} mg/min<br>BW ∈ {75.6, 78.6} kg
Munafo1992 [24]	[Cve_los], [Cve_e3174], [ald]	PODOSE_los ∈ {0, 40, 80, 120} mg<br>BW = 66.5 kg

Table S5. Plotted observables and parameter changes per study simulation (continued). Square brackets around SBML species ids indicate concentrations (amount/volume units). Square brackets enclosing numerical values indicate parameter ranges, whereas curly brackets indicate sets of discrete choices.

StudyID	Plotted (sid)	Changes
Oh2012 [25]	`[Cve_los]`, `[Cve_e3174]`, `mr_e3174_los_plasma`	`PODOSE_los` = 2 mg
Ohtawa1993 [26]	`[Cve_los]`, `Aurine_los`, `[Cve_e3174]`, `Aurine_e3174`, `[ren]`, `[ang2]`, `[ald]`, `SBP`, `DBP`	`PODOSE_los` $\in \{0, 25, 50, 100, 200\}$ mg<br>`BW` = 64.3 kg<br>`ren_ref`, `[ren]` = 10 pg/ml<br>`ang2_ref`, `[ang2]` = 10.3 pg/ml<br>`ald_ref`, `[ald]` = 110.4 pg/ml<br>`SBP_ref` = 116 mmHg<br>`DBP_ref` = 70.5 mmHg
Puris2019 [27]	`[Cve_los]`, `[Cve_e3174]`	`PODOSE_los` = 12.5 mg
Sekino2003 [28]	`mr_e3174_los_plasma`, `mr_e3174_los_urine`, `SBP_change`, `DBP_change`	`PODOSE_los` = 25 mg<br>`BW` $\in \{65.7, 61.7\}$ kg<br>`LI__f_cyp2c9` $\in \{1.0, 0.585\}$
Shin2020 [29]	`[Cve_los]`, `[Cve_e3174]`, `[Cve_los_e3174]`, `Aurine_los_e3174`	`PODOSE_los` = 50 mg<br>`BW` = 67.4 kg<br>`GU__f_abcb1` $\in \{1.0, 0.306, 0.653\}$
Sica1995 [30]	`[Cve_los]`, `Aurine_los`, `[Cve_e3174]`, `Aurine_e3174`	`PODOSE_los` = 100 mg<br>`BW` $\in \{84.6, 75.7, 75.4\}$ kg<br>`KI__f_renal_function` $\in \{0.14, 0.5, 0.95\}$
Tanaka2014 [31]	`[Cve_los]`, `[Cve_e3174]`, `mr_e3174_los_plasma`	`PODOSE_los` = 50 mg
Yasar2002a [32]	`[Cve_los]`, `Aurine_los`, `[Cve_e3174]`, `Aurine_e3174`, `mr_e3174_los_urine`	`PODOSE_los` $\in \{25, 50\}$ mg<br>`LI__f_cyp2c9` $\in \{1.0, 0.17, 0.385, 0.585, 0.6, 0.8\}$

Table S6. Plotted observables and parameter changes per simulation experiment and scan. Square brackets around SBML species ids indicate concentrations (amount/volume units). Square brackets enclosing numerical values indicate parameter ranges, whereas curly brackets indicate sets of discrete choices.

Simulation	Plotted	Changes
DoseDependencyExperiment	`[Cve_los]`, `Aurine_los`, `Afeces_los`, `[Cve_e3174]`, `Aurine_e3174`, `[Cve_l158]`, `[ren]`, `[ang1]`, `[ald]`, `SBP`, `DBP`	`PODOSE_los` $\in [10, 100]$ mg
HepaticRenalImpairment	`[Cve_los]`, `Aurine_los`, `Afeces_los`, `[Cve_e3174]`, `Aurine_e3174`, `[Cve_l158]`, `[ren]`, `[ang1]`, `[ald]`, `SBP`, `DBP`	`PODOSE_los = 50 mg`<br>`KI__f_renal_function` $\in [-1.0, 1.0]$<br>`f_cirrhosis` $\in [0.0, 0.9]$

Table S6. Plotted observables and parameter changes per simulation experiment (continued). Square brackets around SBML species ids indicate concentrations (amount/volume units). Square brackets enclosing numerical values indicate parameter ranges, whereas curly brackets indicate sets of discrete choices.

Simulation	Plotted (sid)	Changes
GeneticPolymorphism	`[Cve_los]`, `Aurine_los`, `Afeces_los`, `[Cve_e3174]`, `Aurine_e3174`, `[Cve_l158]`, `[ren]`, `[ang1]`, `[ald]`, `SBP`, `DBP`	`PODOSE_los = 50 mg`<br>`LI__f_cyp2c9` $\in [-1.0, 1.0]$<br>`GU__f_abcb1` $\in [-1.0, 1.0]$
LosartanParameterScan	`PODOSE_los`, `f_cirrhosis`, `LI__f_cyp2c9`, `GU__f_abcb1`, `KI__f_renal_function`, $\texttt{AUC}_{\texttt{inf}}$, $\texttt{C}_{\texttt{max}}$, `half-life`, $\texttt{SBP}_{\texttt{min}}$, $\texttt{DBP}_{\texttt{min}}$	`PODOSE_los` $\in [10, 100]$ `mg`<br>KI__f_renal_function $\in [-1.0, 1.0]$ (PODOSE_los = 50 mg)<br>f_cirrhosis $\in [0.0, 0.9]$ (PODOSE_los = 50 mg)<br>LI__f_cyp2c9 $\in [-1.0, 1.0]$ (PODOSE_los = 50 mg)<br>`GU__f_abcb1` $\in [-1.0, 1.0]$ (PODOSE_los = 50 mg)

S4. Simulations

S4.1. Azizi1999 (Fig. S2)

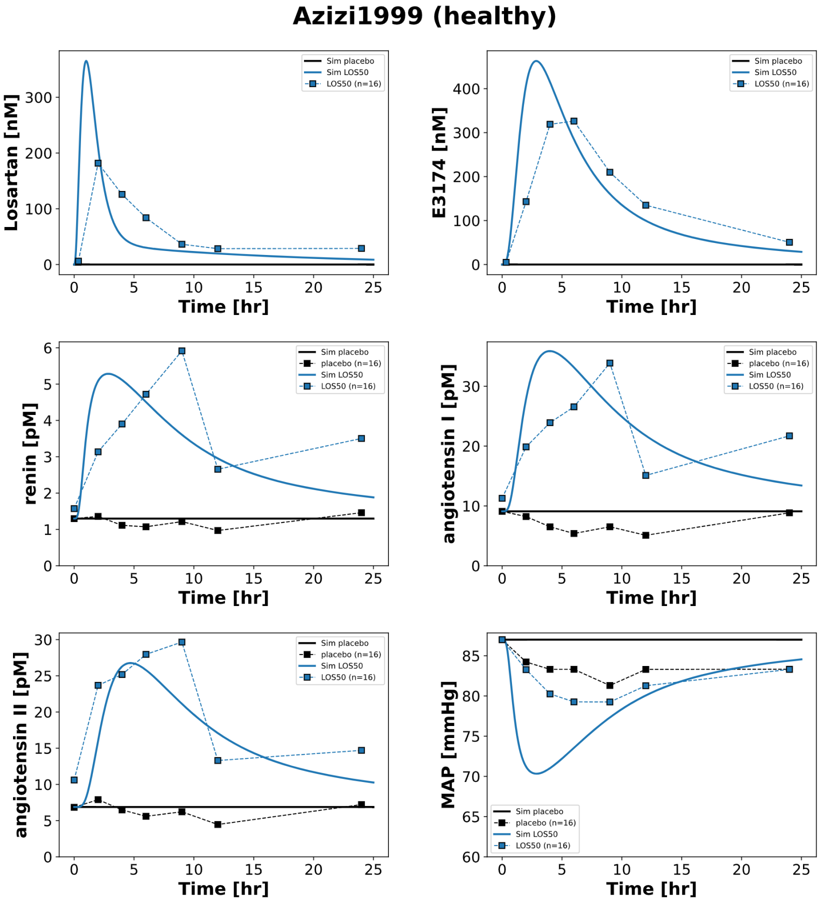

Figure S2. Simulation Azizi1999 [8]. Simulated (solid lines) versus observed (dashed lines with squares) losartan, E3174, renin, angiotensin I, and II plasma concentrations as well as mean arterial pressure (MAP) after placebo or a 50 mg single oral dose of losartan in healthy volunteers.

S4.2. Donzelli2014 (Fig. S3)

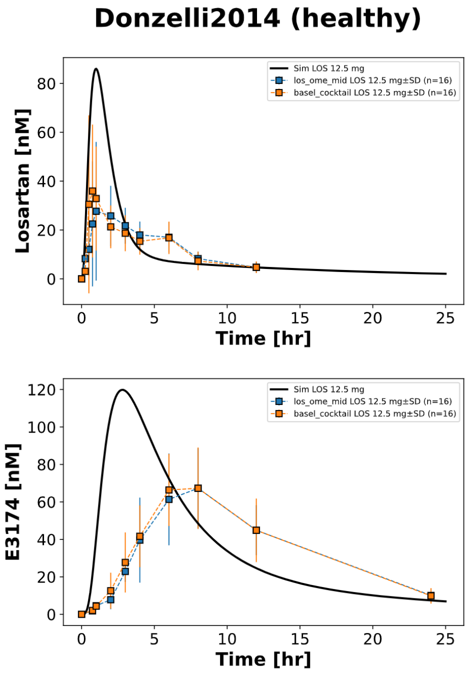

Figure S3. Simulation Donzelli2014 [11]. Simulated (solid lines) versus observed (dashed lines with squares) losartan and E3174 plasma concentrations after a 12.5 mg single oral dose of losartan in healthy volunteers. Administration of the Basel cocktail was not simulated.

S4.3. FDA1995S60 (Fig. S4, S5, S6)

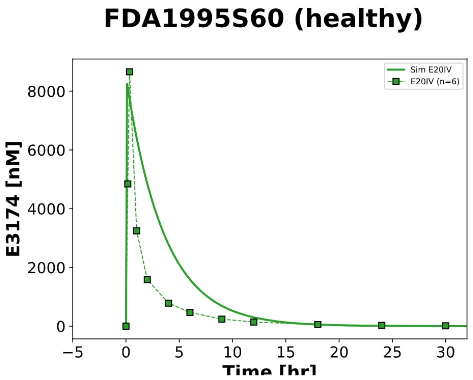

Figure S4. Simulation FDA1995S60 [12]. Simulated (solid lines) versus observed (dashed lines with squares) losartan plasma concentration after a 20 mg single intravenous dose of losartan in healthy volunteers.

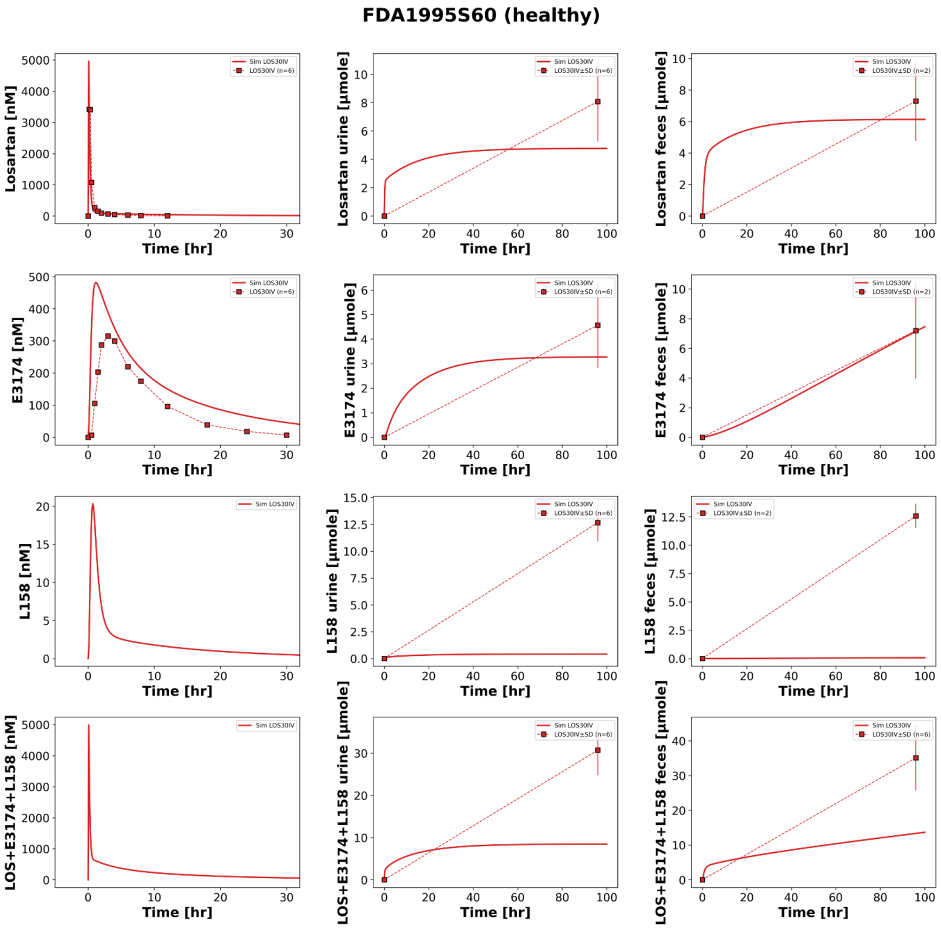

Figure S5. Simulation FDA1995S60 [12]. Simulated (solid lines) versus observed (dashed lines with squares) losartan, E3174, L138 plasma concentrations as well as cumulative amounts of these substances in urine and feces after a 30 mg single intravenous dose of losartan in healthy volunteers.

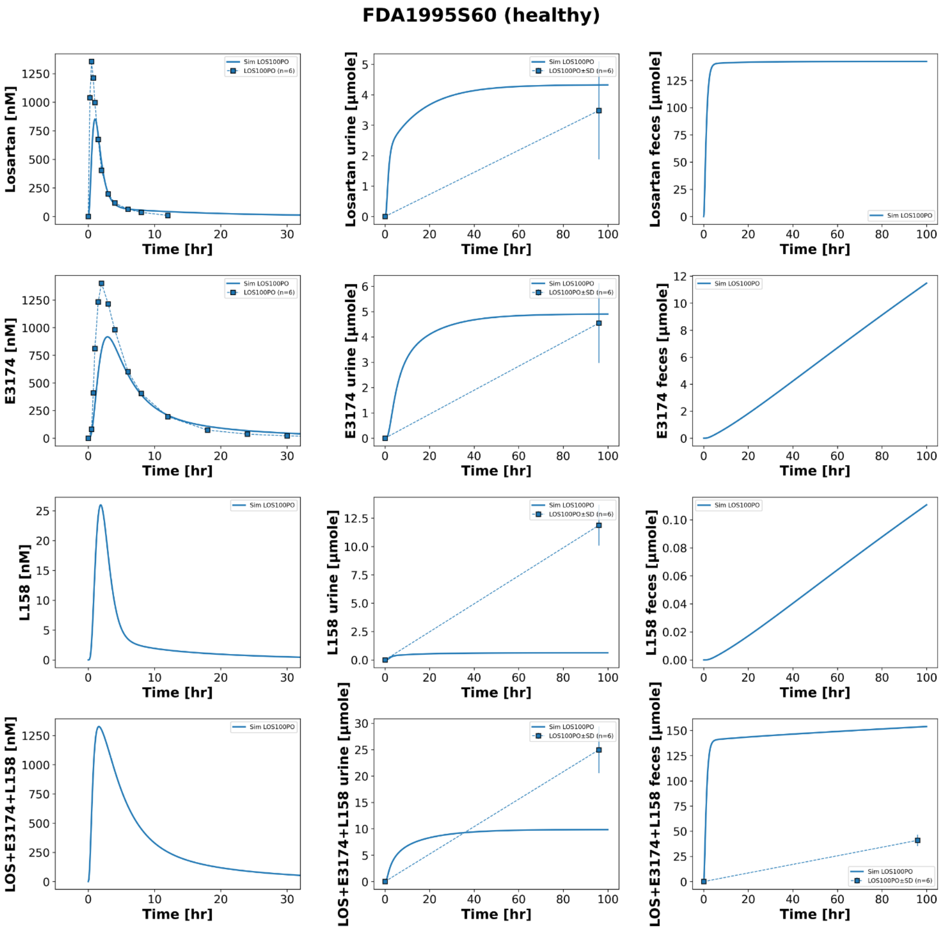

Figure S6. Simulation FDA1995S60 [12]. Simulated (solid lines) versus observed (dashed lines with squares) losartan, E3174, L138 plasma concentrations as well as cumulative amounts of these substances in urine and feces after a 100 mg single oral dose of losartan in healthy volunteers.

S4.4. Fischer2002 (Fig. S7)

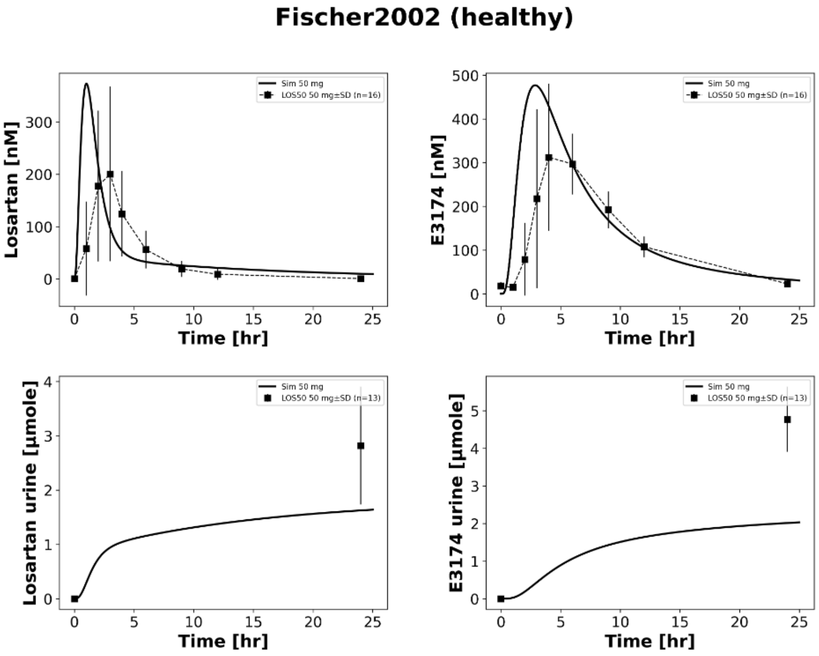

Figure S7. Simulation Fischer2002 [14]. Simulated (solid lines) versus observed (dashed lines with squares) losartan and E3174 plasma concentrations after placebo as well as cumulative amounts of these substances in urine after a 50 mg single oral dose of losartan in healthy volunteers.

S4.5. Goldberg1995 (Fig. S8)

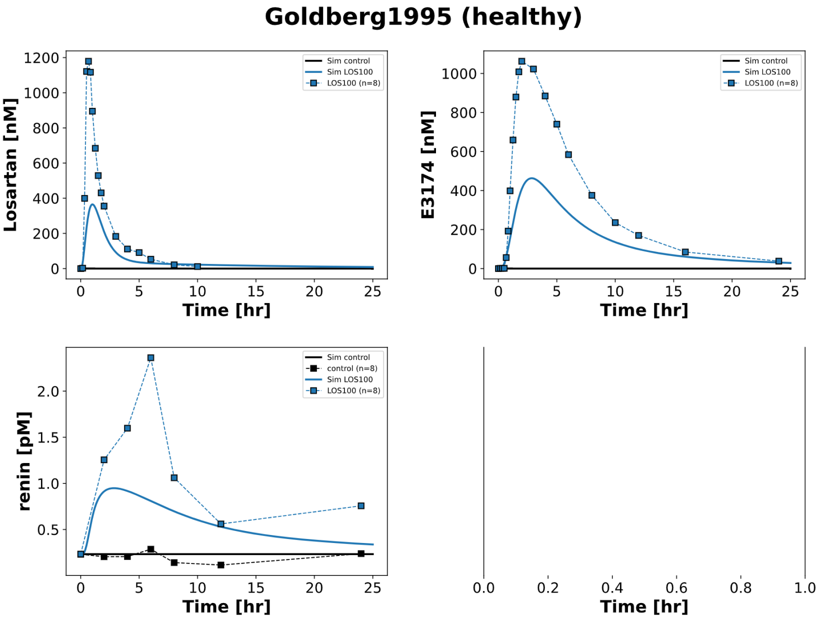

Figure S8. Simulation Goldberg1995 [15]. Simulated (solid lines) versus observed (dashed lines with squares) losartan, E3174, and renin plasma concentrations after placebo or a 50 mg single oral dose of losartan in healthy volunteers.

S4.6. Kim2016 (Fig. S9)

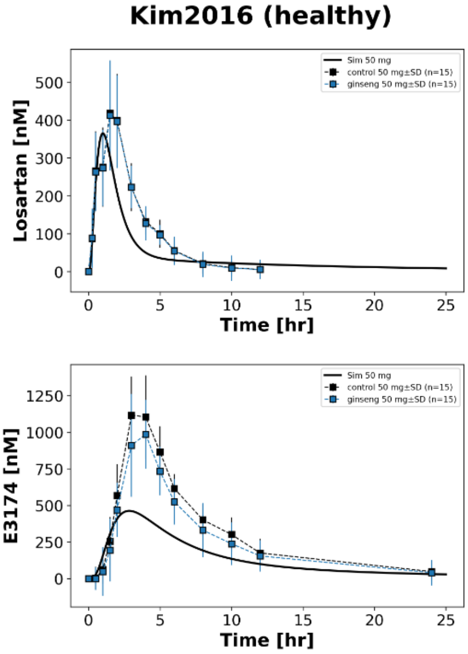

Figure S9. Simulation Kim2016 [19]. Simulated (solid lines) versus observed (dashed lines with squares) losartan and E3174 plasma concentrations after a 50 mg single oral dose of losartan in healthy volunteers.

S4.7. Kobayashi2008 (Fig. S10)

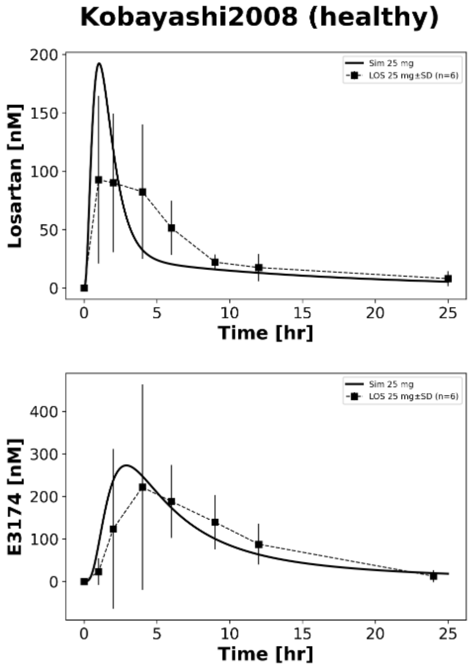

Figure S10. Simulation Kobayashi2008 [20]. Simulated (solid lines) versus observed (dashed lines with squares) losartan and E3174 plasma concentrations after a 25 mg single oral dose of losartan in healthy volunteers.

S4.8. Lo1995 (Fig. S11, S12, S13)

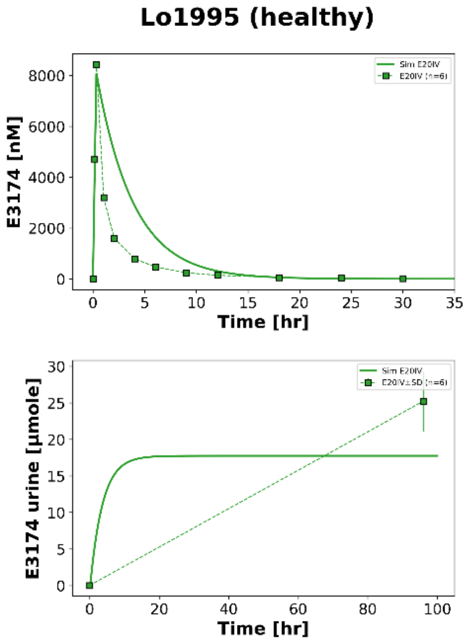

Figure S11. Simulation Lo1995 [23]. Simulated (solid lines) versus observed (dashed lines with squares) E3174 plasma concentration as well as cumulative amount of this substance in urine after a 20 mg single intravenous dose of losartan in healthy volunteers.

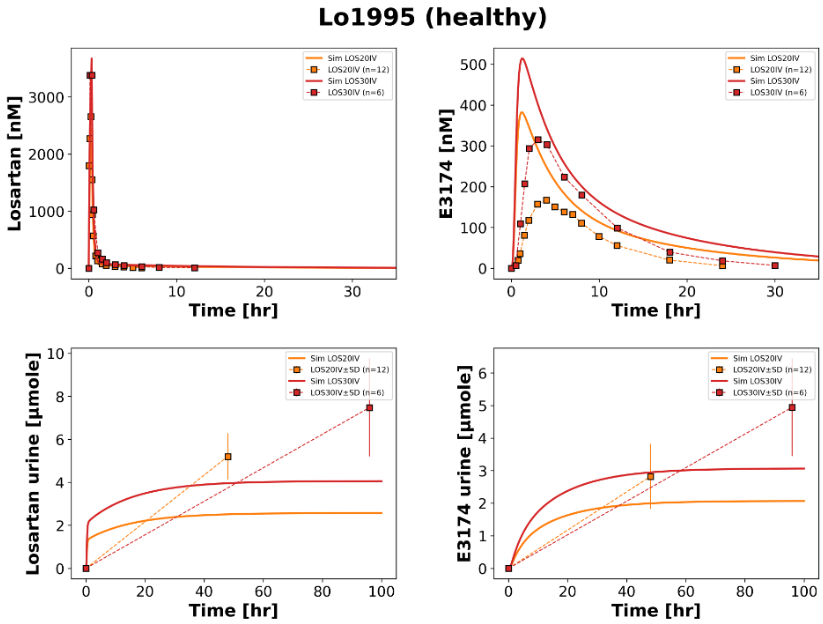

Figure S12. Simulation Lo1995 [23]. Simulated (solid lines) versus observed (dashed lines with squares) losartan and E3174 plasma concentrations, as well as the cumulative amount of these substances in urine after a 20 or 30 mg single intravenous dose of losartan in healthy volunteers.

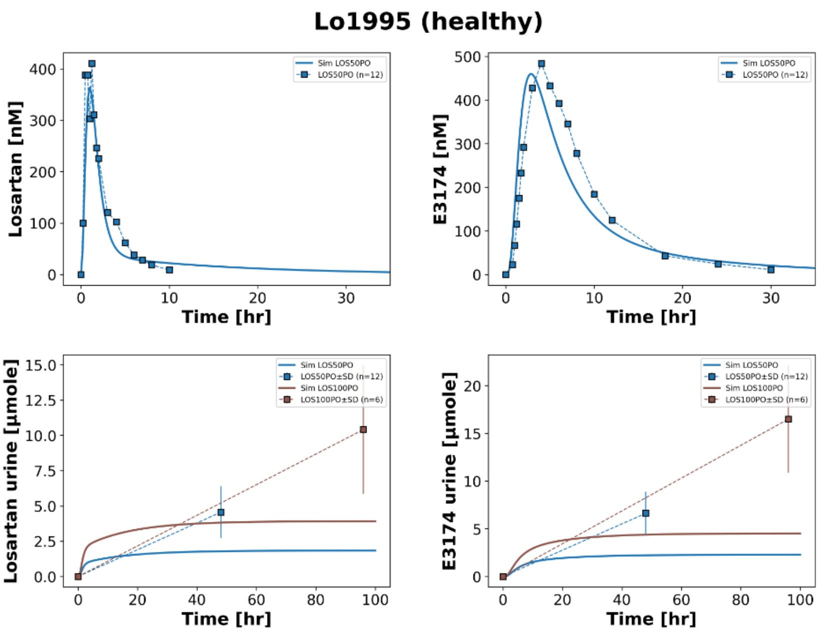

Figure S13. Simulation Lo1995 [23]. Simulated (solid lines) versus observed (dashed lines with squares) losartan and E3174 plasma concentrations, as well as the cumulative amount of these substances in urine after a 50 mg single oral dose of losartan in healthy volunteers.

S4.9. Oh2012 (Fig. S14)

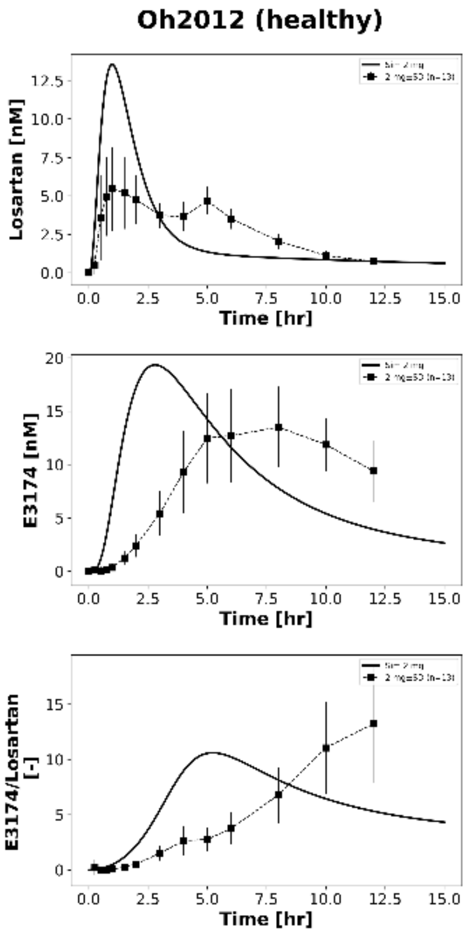

Figure S14. Simulation Oh2012 [25]. Simulated (solid lines) versus observed (dashed lines with squares) losartan, E3174, and L158 plasma concentrations after a 2 mg single oral dose of losartan in healthy volunteers.

S4.10. Puris2019 (Fig. S15)

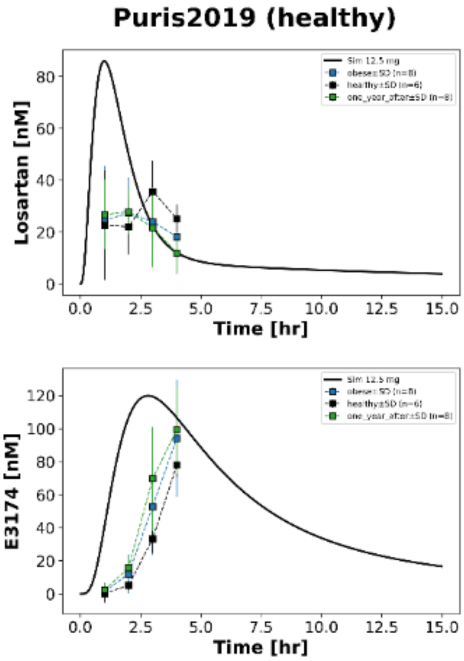

Figure S15. Simulation Puris2019 [27]. Simulated (solid lines) versus observed (dashed lines with squares) losartan and E3174 plasma concentrations after a 12.5 mg single oral dose of losartan in healthy and obese volunteers. The pharmacokinetics of losartan and E3174 for obese volunteers were not simulated.

S4.11. Tanaka2014 (Fig. S16)

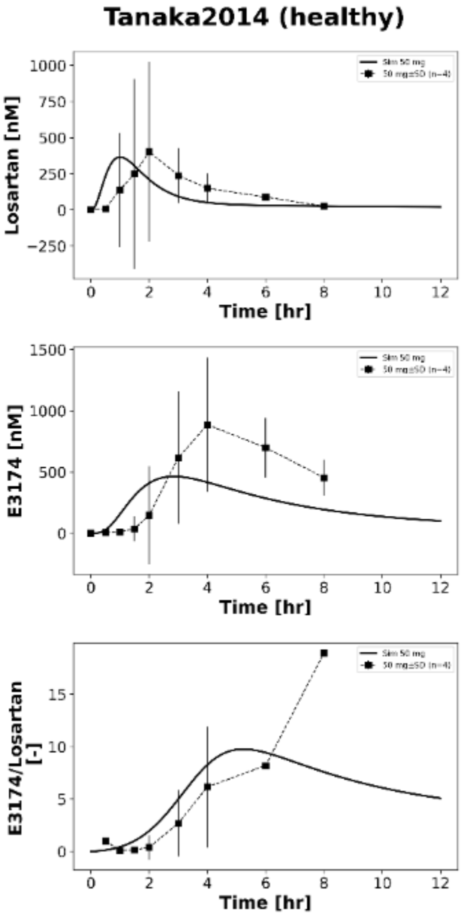

Figure S16. Simulation Tanaka2014 [31]. Simulated (solid lines) versus observed (dashed lines with squares) losartan and E3174 plasma concentrations after a 50 mg single oral dose of losartan in healthy volunteers.

S5. Model Parameters (Tab. S7)

Table S7. Model parameters. ub: upper bound; lb: lower bound

uid	name	value	lb	ub	unit	type	reference
BW	body weight	75	63.8	86.2	kg	data	[33–36]
COBW	cardiac output per bodyweight	1.55	1.32	1.78	ml/s/kg	data	[37–39]
FQgu	gut fractional tissue blood flow	0.18	0.153	0.207	-	data	[34–36]
FQh	hepatic fractional tissue blood flow	0.215	0.183	0.247	-	data	[34–36,40]
FQki	kidney fractional tissue blood flow	0.19	0.162	0.218	-	data	[34–36]
FVar	arterial fractional tissue volume	0.0257	0.0218	0.0296	l/kg	data	[34–36]
FVgu	gut fractional tissue volume	0.0171	0.0145	0.0197	l/kg	data	[34–36]
FVki	kidney fractional tissue volume	0.0044	0.00374	0.00506	l/kg	data	[34–36]
FVli	liver fractional tissue volume	0.021	0.0179	0.0242	l/kg	data	[34–36,40]
FVlu	lung fractional tissue volume	0.0076	0.00646	0.00874	l/kg	data	[34–36]
FVve	venous fractional tissue volume	0.0514	0.0437	0.0591	l/kg	data	[34–36]
GU__F_los_abs	fraction absorbed losartan	0.6	0.51	0.69	-	data	[23]
HCT	hematocrit	0.51	0.433	0.586	-	data	[41,42]
HR	heart rate	70	59.5	80.5	1/min	data	[35,37–39]
LI__LOS2E3174_Km_los	Km losartan → E3174	0.0011	0.000935	0.00127	mmol/l	data	[43–45]
ALDSEC_k	rate aldosterone secretion	1.01e-06	1e-06	1	mmol/min	fitted	
ANGGEN2ANG1_k	rate angen → ang1	0.1	0.01	1e+05	l/min	fitted	
BP_ald_fe	aldosterone effect on blood pressure	0.312	0.1	0.6	-	fitted	
E50_e3174	half-maximum effect E3174	0.000291	5e-07	0.05	mmol/l	fitted	
GU__LOSABS_k	rate of losartan absorption	0.0362	0.0001	1	1/min	fitted	
GU__METEXC_k	rate of feces excretion	2.7e-05	1e-05	0.1	1/min	fitted	
GU__f_LOSEFL_k	fractional rate losartan efflux (PG)	1.02	0.1	10	-	fitted	
KI__E3174EX_k	rate urinary excretion E3174	0.0289	0.0001	1	1/min	fitted	
KI__L158EX_k	rate urinary excretion L158	0.289	0.0001	1	1/min	fitted	
KI__LOSEX_k	rate urinary excretion losartan	0.0774	0.0001	1	1/min	fitted	
Kp_los	tissue/plasma partition coefficient LOS	3.26	1	200	-	fitted	
LI__E3174EX_k	rate E3174 export	0.0112	0.001	10	1/min	fitted	
LI__E3174L158_k	rate losartan → L158	0.00113	1e-05	10	1/min	fitted	
LI__LOS2E3174_Vmax	Vmax losartan → E3174	0.000725	1e-05	100	mmol/min/l	fitted	
LI__MBIEX_k	rate biliary export	0.0663	1e-05	1	1/min	fitted	
ftissue_los	tissue distribution LOS	0.145	0.01	10	l/min	fitted	
GU__Ka_dis_los	rate dissolution losartan	2	1.7	2.3	1/hr	na	
RENSEC_fa_e3174	activation renin secretion by E3174	5	4.25	5.75	-	na	
RENSEC_k	rate renin secretion	0.1	0.085	0.115	mmol/min	na	
GU__f_OATP2B1	absorption activity	1	0.85	1.15	-	scaling	
GU__f_abcb1	PG activity	1	0.85	1.15	-	scaling	[46,47]
KI__f_renal_function	renal function	1	0.85	1.15	-	scaling	[48]
LI__f_cyp2c9	activity of CYP2C9	1	0.85	1.15	-	scaling	[43,45,49]

S6. Parameter Sensitivity Analysis

S6.1. Sampling Sensitivity Analysis (Fig. S17, Tab. S8)

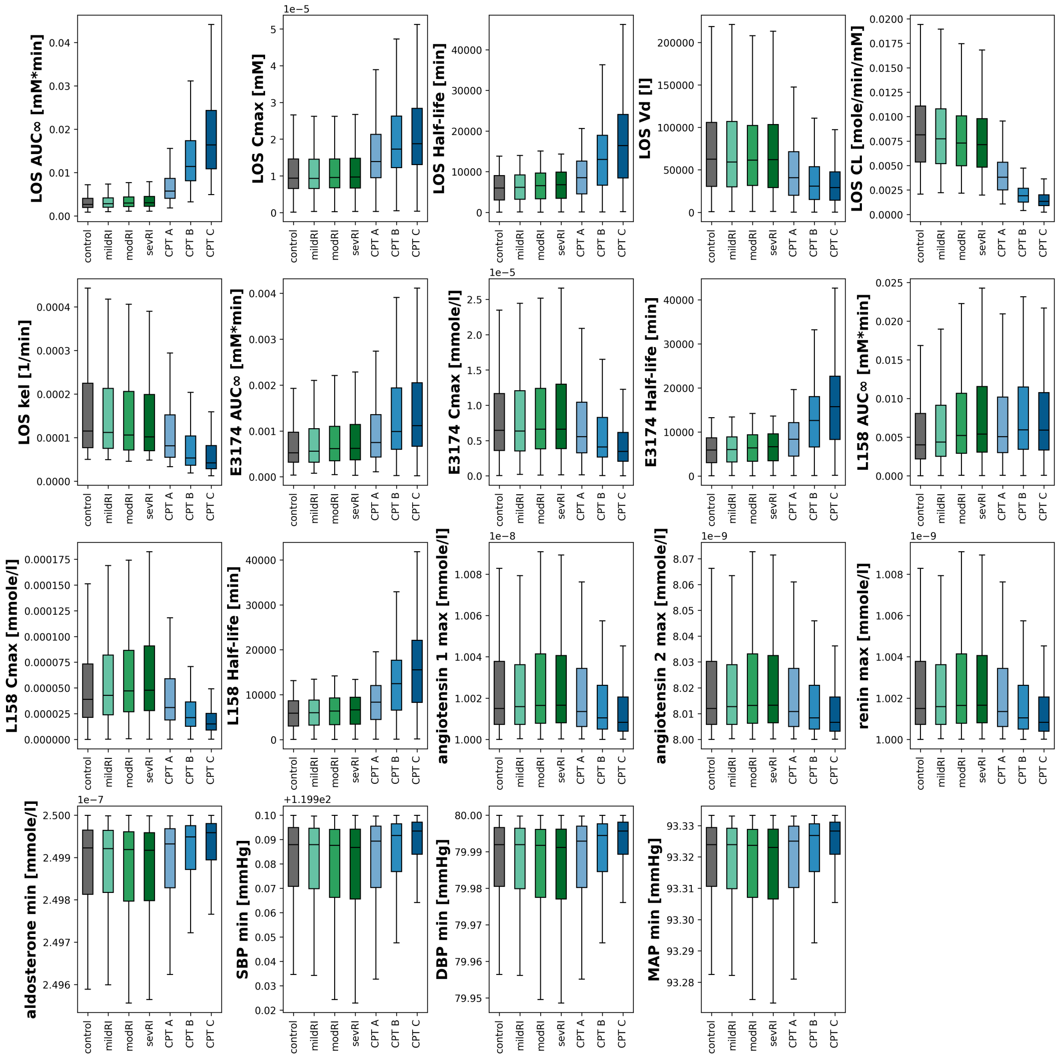

Figure S17. Sampling-based parameter sensitivity. Overview of the influence of parameter uncertainty on pharmacokinetic and pharmacodynamic parameters. The analysis was performed for the following groups: control: healthy control; mildRI: mild renal impairment; modRI: moderate renal impairment; sevRI: severe renal impairment; CPT A: mild cirrhosis; CPT B: moderate cirrhosis; CPT C: severe cirrhosis.

Table S8. Sampling-based parameter sensitivity metrics. Data as mean (coefficient of variation). The analysis was performed for the following groups: control: healthy control; mildRI: mild renal impairment; modRI: moderate renal impairment; sevRI: severe renal impairment; CPT A: mild cirrhosis; CPT B: moderate cirrhosis; CPT C: severe cirrhosis.

	control	mildRI	modRI	sevRI	CPT A	CPT B	CPT C	unit
LOS AUC∞	0.00316 (52.3)	0.00328 (50.4)	0.00349 (51.1)	0.00356 (50.5)	0.00675 (51.5)	0.0137 (57.2)	0.0197 (62.6)	mM·min
LOS Cmax	1.14e-05 (62.0)	1.15e-05 (62.5)	1.16e-05 (62.9)	1.14e-05 (58.7)	1.7e-05 (65.9)	2.1e-05 (60.4)	2.3e-05 (64.2)	mM
LOS Half-life	6.03e+03 (56.8)	6.23e+03 (56.4)	6.54e+03 (56.4)	6.66e+03 (56.0)	8.54e+03 (57.8)	1.31e+04 (59.1)	1.72e+04 (63.1)	min
LOS Vd	7.5e+04 (76.0)	7.4e+04 (77.9)	7.25e+04 (74.6)	7.24e+04 (75.1)	4.96e+04 (76.7)	3.81e+04 (77.2)	3.45e+04 (75.2)	l
LOS CL	0.00873 (47.9)	0.00827 (45.8)	0.0078 (45.5)	0.0076 (45.5)	0.00404 (45.7)	0.00208 (50.3)	0.0015 (53.7)	mole/min/mM
LOS kel	0.000286 (219.0)	0.000273 (225.9)	0.000261 (216.9)	0.00026 (222.8)	0.000202 (225.4)	0.000137 (246.1)	0.000108 (247.4)	1/min
E3174 AUC∞	0.001 (325.1)	0.00101 (168.0)	0.00112 (165.3)	0.00125 (250.8)	0.00135 (203.2)	0.00173 (134.4)	0.00204 (168.3)	mM·min
E3174 Cmax	1.08e-05 (158.6)	1.12e-05 (141.0)	1.2e-05 (156.2)	1.23e-05 (162.9)	9.61e-06 (159.8)	7.08e-06 (117.9)	5.56e-06 (123.1)	mmole/l
E3174 Half-life	5.85e+03 (55.9)	6.05e+03 (55.5)	6.34e+03 (55.4)	6.46e+03 (55.1)	8.26e+03 (56.7)	1.26e+04 (57.4)	1.62e+04 (60.7)	min
L158 AUC∞	0.00775 (157.6)	0.00893 (167.3)	0.0119 (194.6)	0.0141 (257.0)	0.00878 (138.9)	0.00994 (127.9)	0.00964 (137.6)	mM·min
L158 Cmax	6.17e-05 (108.5)	6.55e-05 (106.0)	7.35e-05 (112.3)	7.74e-05 (116.5)	4.61e-05 (97.2)	2.91e-05 (88.1)	1.99e-05 (86.2)	mmole/l
L158 Half-life	5.84e+03 (55.8)	6.03e+03 (55.4)	6.3e+03 (55.2)	6.41e+03 (54.9)	8.22e+03 (56.5)	1.24e+04 (56.9)	1.59e+04 (60.1)	min
angiotensin 1 max	1.01e-08 (12.7)	1.01e-08 (5.8)	1.01e-08 (6.8)	1.01e-08 (4.3)	1.01e-08 (6.9)	1.01e-08 (8.5)	1e-08 (2.8)	mmole/l
angiotensin 2 max	8.1e-09 (12.7)	8.07e-09 (5.8)	8.07e-09 (6.8)	8.07e-09 (4.3)	8.07e-09 (6.9)	8.05e-09 (8.5)	8.03e-09 (2.8)	mmole/l
renin max	1.01e-09 (12.7)	1.01e-09 (5.8)	1.01e-09 (6.8)	1.01e-09 (4.3)	1.01e-09 (6.9)	1.01e-09 (8.5)	1e-09 (2.8)	mmole/l
aldosterone min	2.49e-07 (2.5)	2.5e-07 (1.1)	2.5e-07 (1.4)	2.5e-07 (0.8)	2.5e-07 (1.4)	2.5e-07 (1.8)	2.5e-07 (0.6)	mmole/l
SBP min	120 (1.3)	120 (0.4)	120 (0.5)	120 (0.3)	120 (0.7)	120 (0.9)	120 (0.2)	mmHg
DBP min	79.9 (1.3)	80 (0.4)	79.9 (0.5)	80 (0.3)	79.9 (0.7)	80 (0.9)	80 (0.2)	mmHg
MAP min	93.2 (1.3)	93.3 (0.4)	93.3 (0.5)	93.3 (0.3)	93.3 (0.7)	93.3 (0.9)	93.3 (0.2)	mmHg

S6.2. Local Sensitivity Analysis (Fig. S18)

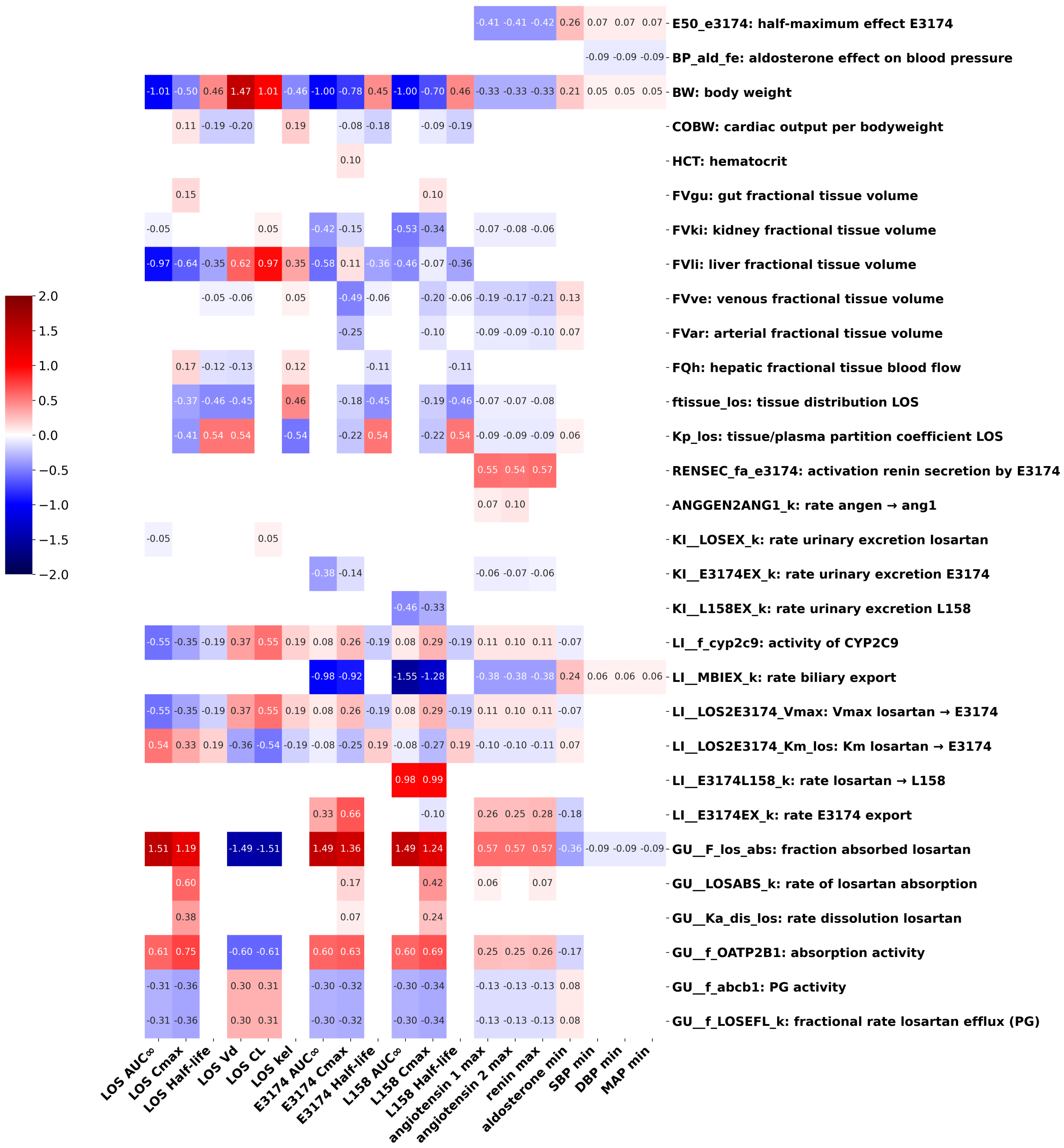

Figure S18. Normalized local parameter sensitivity. Overview of the local influence of parameters on the pharmacokinetic and pharmacodynamic parameters.

S6.3. Global Sensitivity Analysis (Fig. S19, S20)

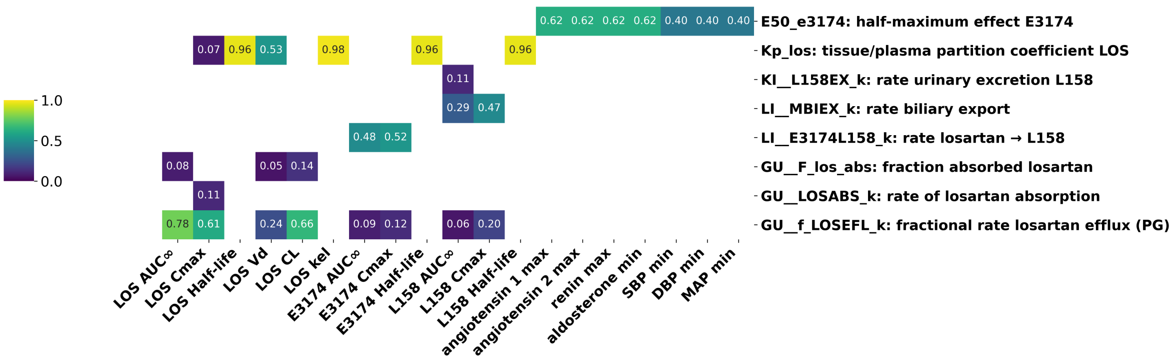

Figure S19. Global parameter sensitivity (Sobol S1). Overview of the global influence of parameters on the pharmacokinetic and pharmacodynamic parameters.

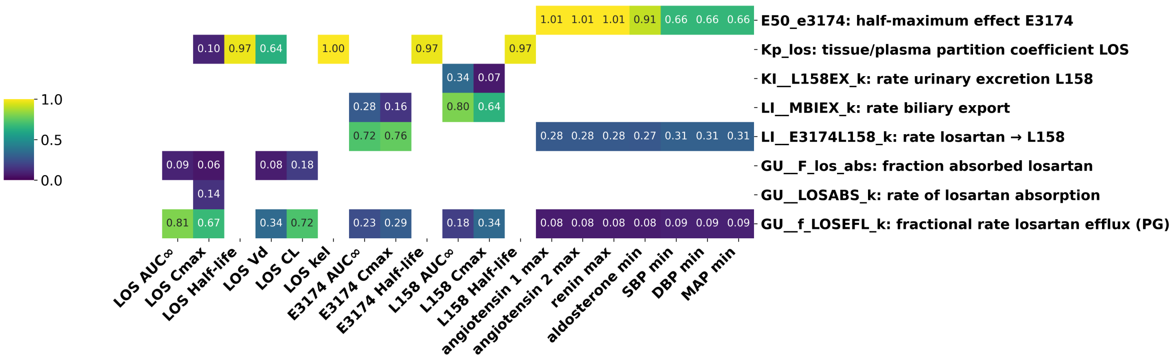

Figure S20. Global parameter sensitivity (Sobol ST). Overview of the global influence of parameters on the pharmacokinetic and pharmacodynamic parameters.

References

1. Babaev, D.; Kutumova, E.; Kolpakov, F. Mathematical Modeling of Pharmacokinetics and Pharmacodynamics of Losartan in Relation to CYP2C9 Allele Variants. *Frontiers in Systems Biology* **2025**, *5*, 1504077. https://doi.org/10.3389/fsysb.2025.1504077.
2. Babaev, D.; Kutumova, E.; Kolpakov, F. Modeling the Influence of CYP2C9 and ABCB1 Gene Polymorphisms on the Pharmacokinetics and Pharmacodynamics of Losartan. *Pharmaceutics* **2025**, *17*, 935. https://doi.org/10.3390/pharmaceutics17070935.
3. Gardiner, P.; Paine, S.W. The Impact of Hepatic Uptake on the Pharmacokinetics of Organic Anions. *Drug metabolism and disposition: the biological fate of chemicals* **2011**, *39*, 1930–1938. https://doi.org/10.1124/dmd.111.039842.
4. Karatza, E.; Karalis, V. Modelling Gastric Emptying: A Pharmacokinetic Model Simultaneously Describing Distribution of Losartan and Its Active Metabolite EXP-3174. *Basic & clinical pharmacology & toxicology* **2020**, *126*, 193–202. https://doi.org/10.1111/bcpt.13321.
5. Karatza, E.; Karalis, V. Investigating the Impact of Gastric Emptying on Pharmacokinetic Parameters Using Delay Differential Equations and Principal Component Analysis. *European journal of drug metabolism and pharmacokinetics* **2021**, *46*, 451–458. https://doi.org/10.1007/s13318-021-00683-3.
6. Nguyen, H.Q.; Lin, J.; Kimoto, E.; Callegari, E.; Tse, S.; Obach, R.S. Prediction of Losartan-Active Carboxylic Acid Metabolite Exposure Following Losartan Administration Using Static and Physiologically Based Pharmacokinetic Models. *Journal of pharmaceutical sciences* **2017**, *106*, 2758–2770. https://doi.org/10.1016/j.xphs.2017.03.032.
7. Ramusovic, S.; Laeer, S. An Integrated Physiology-Based Model for the Interaction of RAA System Biomarkers with Drugs. *Journal of cardiovascular pharmacology* **2012**, *60*, 417–428. https://doi.org/10.1097/FJC.0b013e3182676f06.
8. Azizi, M.; Chatellier, G.; Guyene, T.T.; Ménard, J. Pharmacokinetic-Pharmacodynamic Interactions of Candesartan Cilexetil and Losartan. *Journal of hypertension* **1999**, *17*, 561–568. https://doi.org/10.1097/00004872-199917040-00015.
9. Bae, J.w.; Choi, C.i.; Kim, M.j.; Oh, D.h.; Keum, S.k.; Park, J.i.; Kim, B.h.; Bang, H.k.; Oh, S.g.; Kang, B.s.; et al. Frequency of CYP2C9 Alleles in Koreans and Their Effects on Losartan Pharmacokinetics. *Acta pharmacologica Sinica* **2011**, *32*, 1303–1308. https://doi.org/10.1038/aps.2011.100.
10. Doig, J.K.; MacFadyen, R.J.; Sweet, C.S.; Lees, K.R.; Reid, J.L. Dose-Ranging Study of the Angiotensin Type I Receptor Antagonist Losartan (DuP753/MK954), in Salt-Deplete Normal Man. *Journal of Cardiovascular Pharmacology* **1993**, *21*, 732–738. https://doi.org/10.1097/00005344-199305000-00007.
11. Donzelli, M.; Derungs, A.; Serratore, M.G.; Noppen, C.; Nezic, L.; Krähenbühl, S.; Haschke, M. The Basel Cocktail for Simultaneous Phenotyping of Human Cytochrome P450 Isoforms in Plasma, Saliva and Dried Blood Spots. *Clinical pharmacokinetics* **2014**, *53*, 271–282. https://doi.org/10.1007/s40262-013-0115-0.
12. FDA. FDA1995S60 - 020386Orig1s000rev - COZAAR FDA Review. https://www.accessdata.fda.gov/drugsatfda_docs/nda/96/[illegible] 1995.
13. FDA. FDA1995S67 - 020386Orig1s000rev - COZAAR FDA Review. https://www.accessdata.fda.gov/drugsatfda_docs/nda/96/[illegible] 1995.
14. Fischer, T.L.; Pieper, J.A.; Graff, D.W.; Rodgers, J.E.; Fischer, J.D.; Parnell, K.J.; Goldstein, J.A.; Greenwood, R.; Patterson, J.H. Evaluation of Potential Losartan-Phenytoin Drug Interactions in Healthy Volunteers. *Clinical pharmacology and therapeutics* **2002**, *72*, 238–246. https://doi.org/10.1067/mcp.2002.127945.
15. Goldberg, M.R.; Lo, M.W.; Bradstreet, T.E.; Ritter, M.A.; Höglund, P. Effects of Cimetidine on Pharmacokinetics and Pharmacodynamics of Losartan, an AT1-selective Non-Peptide Angiotensin II Receptor Antagonist. *European journal of clinical pharmacology* **1995**, *49*, 115–119. https://doi.org/10.1007/BF00192369.
16. Goldberg, M.R.; Bradstreet, T.E.; McWilliams, E.J.; Tanaka, W.K.; Lipert, S.; Bjornsson, T.D.; Waldman, S.A.; Osborne, B.; Pivadori, L.; Lewis, G. Biochemical Effects of Losartan, a Nonpeptide Angiotensin II Receptor Antagonist, on the Renin-Angiotensin-Aldosterone System in Hypertensive Patients. *Hypertension (Dallas, Tex.: 1979)* **1995**, *25*, 37–46. https://doi.org/10.1161/01.hyp.25.1.37.
17. Han, Y.; Guo, D.; Chen, Y.; Chen, Y.; Tan, Z.R.; Zhou, H.H. Effect of Silymarin on the Pharmacokinetics of Losartan and Its Active Metabolite E-3174 in Healthy Chinese Volunteers. *European journal of clinical pharmacology* **2009**, *65*, 585–591. https://doi.org/10.1007/s00228-009-0624-9.
18. Huang, H.X.; Wu, H.; Zhao, Y.; Zhou, T.; Ai, X.; Dong, Y.; Zhang, Y.; Lai, Y. Effect of CYP2C9 Genetic Polymorphism and Breviscapine on Losartan Pharmacokinetics in Healthy Subjects. *Xenobiotica; the fate of foreign compounds in biological systems* **2021**, *51*, 616–623. https://doi.org/10.1080/00498254.2021.1880670.

19. Kim, M.G.; Kim, Y.; Jeon, J.Y.; Kim, D.S. Effect of Fermented Red Ginseng on Cytochrome P450 and P-glycoprotein Activity in Healthy Subjects, as Evaluated Using the Cocktail Approach. *British journal of clinical pharmacology* **2016**, *82*, 1580–1590. https://doi.org/10.1111/bcp.13080.
20. Kobayashi, M.; Takagi, M.; Fukumoto, K.; Kato, R.; Tanaka, K.; Ueno, K. The Effect of Bucolome, a CYP2C9 Inhibitor, on the Pharmacokinetics of Losartan. *Drug metabolism and pharmacokinetics* **2008**, *23*, 115–119. https://doi.org/10.2133/dmpk.23.115.
21. Lee, C.R.; Pieper, J.A.; Hinderliter, A.L.; Blaisdell, J.A.; Goldstein, J.A. Losartan and E3174 Pharmacokinetics in Cytochrome P450 2C9*1/*1, *1/*2, and *1/*3 Individuals. *Pharmacotherapy* **2003**, *23*, 720–725. https://doi.org/10.1592/phco.23.6.720.32187.
22. Li, Z.; Wang, G.; Wang, L.S.; Zhang, W.; Tan, Z.R.; Fan, L.; Chen, B.L.; Li, Q.; Liu, J.; Tu, J.H.; et al. Effects of the CYP2C9*13 Allele on the Pharmacokinetics of Losartan in Healthy Male Subjects. *Xenobiotica; the fate of foreign compounds in biological systems* **2009**, *39*, 788–793. https://doi.org/10.1080/00498250903134435.
23. Lo, M.W.; Goldberg, M.R.; McCrea, J.B.; Lu, H.; Furtek, C.I.; Bjornsson, T.D. Pharmacokinetics of Losartan, an Angiotensin II Receptor Antagonist, and Its Active Metabolite EXP3174 in Humans. *Clinical pharmacology and therapeutics* **1995**, *58*, 641–649. https://doi.org/10.1016/0009-9236(95)90020-9.
24. Munafo, A.; Christen, Y.; Nussberger, J.; Shum, L.Y.; Borland, R.M.; Lee, R.J.; Waeber, B.; Biollaz, J.; Brunner, H.R. Drug Concentration Response Relationships in Normal Volunteers after Oral Administration of Losartan, an Angiotensin II Receptor Antagonist. *Clinical pharmacology and therapeutics* **1992**, *51*, 513–521. https://doi.org/10.1038/clpt.1992.56.
25. Oh, K.S.; Park, S.J.; Shinde, D.D.; Shin, J.G.; Kim, D.H. High-Sensitivity Liquid Chromatography-Tandem Mass Spectrometry for the Simultaneous Determination of Five Drugs and Their Cytochrome P450-specific Probe Metabolites in Human Plasma. *Journal of chromatography. B, Analytical technologies in the biomedical and life sciences* **2012**, *895–896*, 56–64. https://doi.org/10.1016/j.jchromb.2012.03.014.
26. Ohtawa, M.; Takayama, F.; Saitoh, K.; Yoshinaga, T.; Nakashima, M. Pharmacokinetics and Biochemical Efficacy after Single and Multiple Oral Administration of Losartan, an Orally Active Nonpeptide Angiotensin II Receptor Antagonist, in Humans. *British journal of clinical pharmacology* **1993**, *35*, 290–297. https://doi.org/10.1111/j.1365-2125.1993.tb05696.x.
27. Puris, E.; Pasanen, M.; Ranta, V.P.; Gynther, M.; Petsalo, A.; Käkelä, P.; Männistö, V.; Pihlajamäki, J. Laparoscopic Roux-en-Y Gastric Bypass Surgery Influenced Pharmacokinetics of Several Drugs given as a Cocktail with the Highest Impact Observed for CYP1A2, CYP2C8 and CYP2E1 Substrates. *Basic & clinical pharmacology & toxicology* **2019**, *125*, 123–132. https://doi.org/10.1111/bcpt.13234.
28. Sekino, K.; Kubota, T.; Okada, Y.; Yamada, Y.; Yamamoto, K.; Horiuchi, R.; Kimura, K.; Iga, T. Effect of the Single CYP2C9*3 Allele on Pharmacokinetics and Pharmacodynamics of Losartan in Healthy Japanese Subjects. *European journal of clinical pharmacology* **2003**, *59*, 589–592. https://doi.org/10.1007/s00228-003-0664-5.
29. Shin, H.B.; Jung, E.H.; Kang, P.; Lim, C.W.; Oh, K.Y.; Cho, C.K.; Lee, Y.J.; Choi, C.I.; Jang, C.G.; Lee, S.Y.; et al. ABCB1 c.2677G>T/c.3435C>T Diplotype Increases the Early-Phase Oral Absorption of Losartan. *Archives of pharmacal research* **2020**, *43*, 1187–1196. https://doi.org/10.1007/s12272-020-01294-3.
30. Sica, D.A.; Lo, M.W.; Shaw, W.C.; Keane, W.F.; Gehr, T.W.; Halstenson, C.E.; Lipschutz, K.; Furtek, C.I.; Ritter, M.A.; Shahinfar, S. The Pharmacokinetics of Losartan in Renal Insufficiency. *Journal of hypertension. Supplement : official journal of the International Society of Hypertension* **1995**, *13*, S49–52. https://doi.org/10.1097/00004872-199507001-00007.
31. Tanaka, S.; Uchida, S.; Inui, N.; Takeuchi, K.; Watanabe, H.; Namiki, N. Simultaneous LC-MS/MS Analysis of the Plasma Concentrations of a Cocktail of 5 Cytochrome P450 Substrate Drugs and Their Metabolites. *Biological & pharmaceutical bulletin* **2014**, *37*, 18–25. https://doi.org/10.1248/bpb.b13-00401.
32. Yasar, U.; Dahl, M.L.; Christensen, M.; Eliasson, E. Intra-Individual Variability in Urinary Losartan Oxidation Ratio, an in Vivo Marker of CYP2C9 Activity. *British journal of clinical pharmacology* **2002**, *54*, 183–185. https://doi.org/10.1046/j.1365-2125.2002.01646.x.
33. Ogden, C.L.; Fryar, C.D.; Carroll, M.D.; Flegal, K.M. Mean Body Weight, Height, and Body Mass Index, United States 1960-2002. *Advance Data* **2004**, pp. 1–17.
34. Jones, H.; Rowland-Yeo, K. Basic Concepts in Physiologically Based Pharmacokinetic Modeling in Drug Discovery and Development. *CPT: Pharmacometrics & Systems Pharmacology* **2013**, *2*, 1–12. https://doi.org/10.1038/psp.2013.41.
35. Thompson, C.M.; Johns, D.O.; Sonawane, B.; Barton, H.A.; Hattis, D.; Tardif, R.; Krishnan, K. Database for Physiologically Based Pharmacokinetic (PBPK) Modeling: Physiological Data for Healthy and Health-

Impaired Elderly. *Journal of Toxicology and Environmental Health. Part B, Critical Reviews* **2009**, *12*, 1–24. https://doi.org/10.1080/10937400802545060.

36. Brown, R.P.; Delp, M.D.; Lindstedt, S.L.; Rhomberg, L.R.; Beliles, R.P. Physiological Parameter Values for Physiologically Based Pharmacokinetic Models. *Toxicology and Industrial Health* **1997**, *13*, 407–484. https://doi.org/10.1177/074823379701300401.
37. Cattermole, G.N.; Leung, P.Y.M.; Ho, G.Y.L.; Lau, P.W.S.; Chan, C.P.Y.; Chan, S.S.W.; Smith, B.E.; Graham, C.A.; Rainer, T.H. The Normal Ranges of Cardiovascular Parameters Measured Using the Ultrasonic Cardiac Output Monitor. *Physiological Reports* **2017**, *5*, e13195. https://doi.org/10.14814/phy2.13195.
38. Patel, H.N.; Miyoshi, T.; Addetia, K.; Henry, M.P.; Citro, R.; Daimon, M.; Gutierrez Fajardo, P.; Kasliwal, R.R.; Kirkpatrick, J.N.; Monaghan, M.J.; et al. Normal Values of Cardiac Output and Stroke Volume According to Measurement Technique, Age, Sex, and Ethnicity: Results of the World Alliance of Societies of Echocardiography Study. *Journal of the American Society of Echocardiography: Official Publication of the American Society of Echocardiography* **2021**, *34*, 1077–1085.e1. https://doi.org/10.1016/j.echo.2021.05.012.
39. Collis, T.; Devereux, R.B.; Roman, M.J.; De Simone, G.; Yeh, J.L.; Howard, B.V.; Fabsitz, R.R.; Welty, T.K. Relations of Stroke Volume and Cardiac Output to Body Composition: The Strong Heart Study. *Circulation* **2001**, *103*, 820–825. https://doi.org/10.1161/01.CIR.103.6.820.
40. Wynne, H.A.; Cope, L.H.; Mutch, E.; Rawlins, M.D.; Woodhouse, K.W.; James, O.F. The Effect of Age upon Liver Volume and Apparent Liver Blood Flow in Healthy Man. *Hepatology (Baltimore, Md.)* **1989**, *9*, 297–301. https://doi.org/10.1002/hep.1840090222.
41. Mondal, H.; Zubair, M. Hematocrit. In *StatPearls*; StatPearls Publishing: Treasure Island (FL), 2025.
42. Fiseha, T.; Alemayehu, E.; Mohammed, O.; Gedefie, A.; Adamu, A.; Tamir, Z.; Gebreweld, A. Reference Intervals of Haematological Parameters for Apparently Healthy Adults in Northeast Ethiopia. *International Journal of General Medicine* **2023**, *16*, 5309–5321. https://doi.org/10.2147/IJGM.S430751.
43. Maekawa, K.; Harakawa, N.; Sugiyama, E.; Tohkin, M.; Kim, S.R.; Kaniwa, N.; Katori, N.; Hasegawa, R.; Yasuda, K.; Kamide, K.; et al. Substrate-Dependent Functional Alterations of Seven CYP2C9 Variants Found in Japanese Subjects. *Drug Metabolism and Disposition: The Biological Fate of Chemicals* **2009**, *37*, 1895–1903. https://doi.org/10.1124/dmd.109.027003.
44. Thu, O.K.F.; Spigset, O.; Hellum, B. Noncompetitive Inhibition of Human CYP2C9 in Vitro by a Commercial Rhodiola Rosea Product. *Pharmacology Research & Perspectives* **2017**, *5*, e00324. https://doi.org/10.1002/prp2.324.
45. Wang, Y.H.; Pan, P.P.; Dai, D.P.; Wang, S.H.; Geng, P.W.; Cai, J.P.; Hu, G.X. Effect of 36 CYP2C9 Variants Found in the Chinese Population on Losartan Metabolism in Vitro. *Xenobiotica; the fate of foreign compounds in biological systems* **2014**, *44*, 270–275. https://doi.org/10.3109/00498254.2013.820007.
46. Siegmund, W.; Ludwig, K.; Giessmann, T.; Dazert, P.; Schroeder, E.; Sperker, B.; Warzok, R.; Kroemer, H.K.; Cascorbi, I. The Effects of the Human MDR1 Genotype on the Expression of Duodenal P-glycoprotein and Disposition of the Probe Drug Talinolol. *Clinical Pharmacology and Therapeutics* **2002**, *72*, 572–583. https://doi.org/10.1067/mcp.2002.127739.
47. Hoffmeyer, S.; Burk, O.; von Richter, O.; Arnold, H.P.; Brockmöller, J.; Johne, A.; Cascorbi, I.; Gerloff, T.; Roots, I.; Eichelbaum, M.; et al. Functional Polymorphisms of the Human Multidrug-Resistance Gene: Multiple Sequence Variations and Correlation of One Allele with P-glycoprotein Expression and Activity in Vivo. *Proceedings of the National Academy of Sciences of the United States of America* **2000**, *97*, 3473–3478. https://doi.org/10.1073/pnas.97.7.3473.
48. Stevens, P.E.; Ahmed, S.B.; Carrero, J.J.; Foster, B.; Francis, A.; Hall, R.K.; Herrington, W.G.; Hill, G.; Inker, L.A.; Kazancıoğlu, R.; et al. KDIGO 2024 Clinical Practice Guideline for the Evaluation and Management of Chronic Kidney Disease. *Kidney International* **2024**, *105*, S117–S314. https://doi.org/10.1016/j.kint.2023.10.018.
49. Kusama, M.; Maeda, K.; Chiba, K.; Aoyama, A.; Sugiyama, Y. Prediction of the Effects of Genetic Polymorphism on the Pharmacokinetics of CYP2C9 Substrates from in Vitro Data. *Pharmaceutical research* **2009**, *26*, 822–835. https://doi.org/10.1007/s11095-008-9781-2.
